# Seasonal and geographical differences in the ruminal microbial and chloroplast composition of sika deer (*Cervus nippon*) in Japan

**DOI:** 10.1038/s41598-022-09855-w

**Published:** 2022-04-15

**Authors:** Shinpei Kawarai, Kensuke Taira, Ayako Shimono, Tsuyoshi Takeshita, Shiro Takeda, Wataru Mizunoya, Yumiko Yamazaki, Shigeharu Moriya, Masato Minami

**Affiliations:** 1 Laboratory of Small Animal Clinics, Veterinary Teaching Hospital, Azabu University, 1-17-71 Fuchinobe, Chuo-ku, Sagamihara, Kanagawa 252-5201 Japan; 2 Laboratory of Parasitology, School of Veterinary Medicine, Azabu University, 1-17-71 Fuchinobe, Chuo-ku, Sagamihara, Kanagawa 252-5201 Japan; 3 Plant Ecology Laboratory, Department of Biology, Faculty of Science, Toho University, 2-2-1 Miyama, Funabashi-shi, Chiba 274-8510 Japan; 4 Agriculture and Forestry Division, Komoro City Office, 3-3-3, Aioicho, Komoro, Nagano 384-8501 Japan; 5 Laboratory of Food Science, Department of Animal Science and Biotechnology, School of Veterinary Medicine, Azabu University, 1-17-71 Fuchinobe, Chuo-ku, Sagamihara, Kanagawa 252-5201 Japan; 6 RIKEN BDR, Kobe, Hyogo 650-0047 Japan; 7 RIKEN CSRS, Yokohama, Kanagawa 230-0045 Japan; 8 Laboratory of Wildlife Ecology and Conservation, Department of Animal Science and Biotechnology, School of Veterinary Medicine, Azabu University, 1-17-71 Fuchinobe, Chuo-ku, Sagamihara, Kanagawa 252-5201 Japan; 9 Center for Human and Animal Symbiosis Science, Azabu University, 1-17-71 Fuchinobe, Chuo-ku, Sagamihara, Kanagawa 252-5201 Japan

**Keywords:** Microbial communities, Transcriptomics

## Abstract

To understand the nutritional status of culled wild sika deer (*Cervus nippon*), we compared the ruminal microbes of deer living in habitats differing in food composition (Nagano winter, Nagano spring, and Hokkaido winter) using next-generation sequencing. Twenty-nine sika deer were sampled. Alpha and beta diversity metrics determined via 16S and 18S rRNA amplicon-seq analysis showed compositional differences. *Prevotella*, *Entodinium*, and *Piromyces* were the dominant genera of bacteria, fungi and protozoa, respectively. Moreover, 66 bacterial taxa, 44 eukaryotic taxa, and 46 chloroplastic taxa were shown to differ significantly among the groups by the linear discriminant analysis effect size (LEfSe) technique. Total RNA-seq analysis yielded 397 significantly differentially expressed transcripts (q < 0.05), of which 48 (q < 0.01) were correlated with the bacterial amplicon-seq results (Pearson correlation coefficient > 0.7). The ruminal microbial composition corresponded with the presence of different plants because the amplicon-seq results indicated that chloroplast from broadleaf trees and Stramenopiles-Alveolates-Rhizaria (SAR) were enriched in Nagano, whereas chloroplast from graminoids, Firmicutes and the dominant phylum of fungi were enriched in Hokkaido. These results could be related to the severe snow conditions in Hokkaido in winter and the richness of plants with leaves and acorns in Nagano in winter and spring. The findings are useful for understanding the nutritional status of wild sika deer.

## Introduction

In Japan, the extinction of apex predator wolves, global warming, reduced snowfall, and the declining popularity of hunting increased the number of sika deer (*Cervus nippon*) approximately tenfold from 1990 to 2014, with the population estimated to be 3.05 million animals^1^. Because deer feed on plants for survival, an excess of deer disturbs forest ecosystems, damages crops, and increases the prevalence of tick-borne diseases in humans in cities^1,2^. The Japanese government has tried to control the deer population by culling and using deer resources for some purposes, such as “game meat” for human consumption^1^. However, the high cost of disposing of carcasses is an economic burden on local authorities^3^. To solve this problem, the use of deer resources for pet food has recently been attempted to cover facility costs^3^.

The rumen, the digestive organ of ruminants, contains microbes that ferment plant cellulose by producing volatile fatty acids (VFAs) and methane as an energy source^4,5^. The rumen flora is composed of bacteria, archaea, protozoa, fungi, and viruses, and they work collaboratively to maintain the nutritional homeostasis of the host^4–6^. The rumen microbial diversity of wild deer has been studied to understand rumen development over the long history of adaptation to natural habitats^7^. The differences in rumen species caused by living locations and seasons have been studied in reindeer^7–9^ and sika deer^10,11^. However, the influence of food habits on the ruminal community in wild deer remains unexplored^12,13^, especially in Japan^11^.

Recently, high-throughput 16S and 18S small subunit ribosomal RNA (rRNA) gene sequencing (amplicon-seq) has become available for the analysis of rumen microbial community diversity^12–19^. Studies have reported species differences among red deer, roe deer, and moose^12^ and in association with diet in livestock and wild deer^13^. A few amplicon-seq studies have examined the effect on rumen bacterial, fungal, and protozoal composition in wild deer. In one study. Li et al.^17^ reported that tannin-rich plants altered the rumen microbiome and fermentation patterns in captive sika deer in China. Wilson et al.^13^ reported an association between the diet and rumen microbiota in wild roe deer, but protozoa were observed in only 1% of the population and were unevenly distributed. These studies examined the factors that drive changes in rumen microbial diversity. Thus, a knowledge of ruminal microbes in relation to plant compositions is important for understanding nutritional status and growth.

To promote the use of culled sika deer for human and companion animal consumption in Japan, we were interested in examining the rumen microbial diversity of sika deer, including bacteria, eukarya, and chloroplast composition, to understand host nutritional status. To identify the factors affecting the quality of wild deer meat, it is necessary to collect more information on wild deer biology and ecology. In general, the amount and type of feed eaten by wild deer depend on the location and season. Feed intake increases in spring and reaches a peak in summer^21^. Stanisz et al.^22^ reported that deer meat obtained in winter was brighter and less red than that obtained in summer. Thus, seasonal variation in food habits is likely an important determinant of deer meat quality. Nonetheless, little information about the relationship between food habits and meat quality is available.

Many wild sika deer inhabit Mount Asama, Nagano, the central northern area of Honshu Island (36′ N, 139′ E), and Shiretoko, the northernmost island of Hokkaido (44′ N, 145′ E), Japan. In Shiretoko, the winter is long and cold (with temperatures below zero) with heavy snowfall, so sika deer experience limited access to food^10,23^. In Nagano, as reported in Ashio, Tochigi (36′ N, 139′ E)^24^, and Akagi, Gunma (36′ N, 139′ E)^25^, oak forests and conifer stands are dominant, and sika deer can obtain much more food in winter. Similar to the findings of a study in Poland^22^, it is possible that geographical and seasonal variations in food habits affect meat quality in Japanese sika deer.

Total RNA sequencing (RNA-seq) aims to describe microbial ecology and the transcriptome simultaneously. It has been widely used in studies of marine ecosystems^26,27^, soil microbes^28,29^ and rumen microbiomes^30,31^ to obtain information from all domains of microbial life, including eukaryotes, archaea, and bacteria. In particular, unlike the genomic DNA sequencing approach, this RNA-based transcriptome approach can describe differences in functional gene expression patterns among live samples.

In this study, we examined the effects of season (spring/winter) and location (Nagano/Hokkaido) on the population-level microbiome and transcriptome diversity in wild sika deer by using 16S and 18S rRNA amplicon-seq and total RNA-seq analysis.

## Results

### Richness and evenness of ruminal microbial and chloroplast composition among sampling seasons and locations revealed using amplicon-seq

For amplicon-seq, raw read data were obtained from 29 deer [12 Nagano winter (NW) deer, 12 Nagano spring (NS) deer, and 5 Hokkaido winter (HW) deer]. Two read datasets, both from NS deer, were discarded because too few reads met the quality filtering criteria. The profiles of 27 sika deer that were enrolled and 2 that were excluded based on quality checks in this study are shown in Supplementary Tables S1 and S2. The total read count (mean read count per sample) of this study was 7,042,740 (260,842 ± 87,194 SD), 6,387,893 (236,588 ± 138,486 SD), and 2,151,717 (79,693 ± 75,712 SD) for Bacterial 16S rRNA (Bac), Eukaryal 18S rRNA without chloroplasts and host mitochondria (Euk), and Chloroplast 18S rRNA (Chl) gene sequences, respectively. The total numbers of operational taxonomic units (OTUs) (mean OTUs observed per sample) identified in this study were 10,055 (372 ± 42 SD) Bac OTUs, 1252 (46 ± 14 SD) Euk OTUs and 1006 (37 ± 12 SD) Chl OTUs.

**Figure 1:**
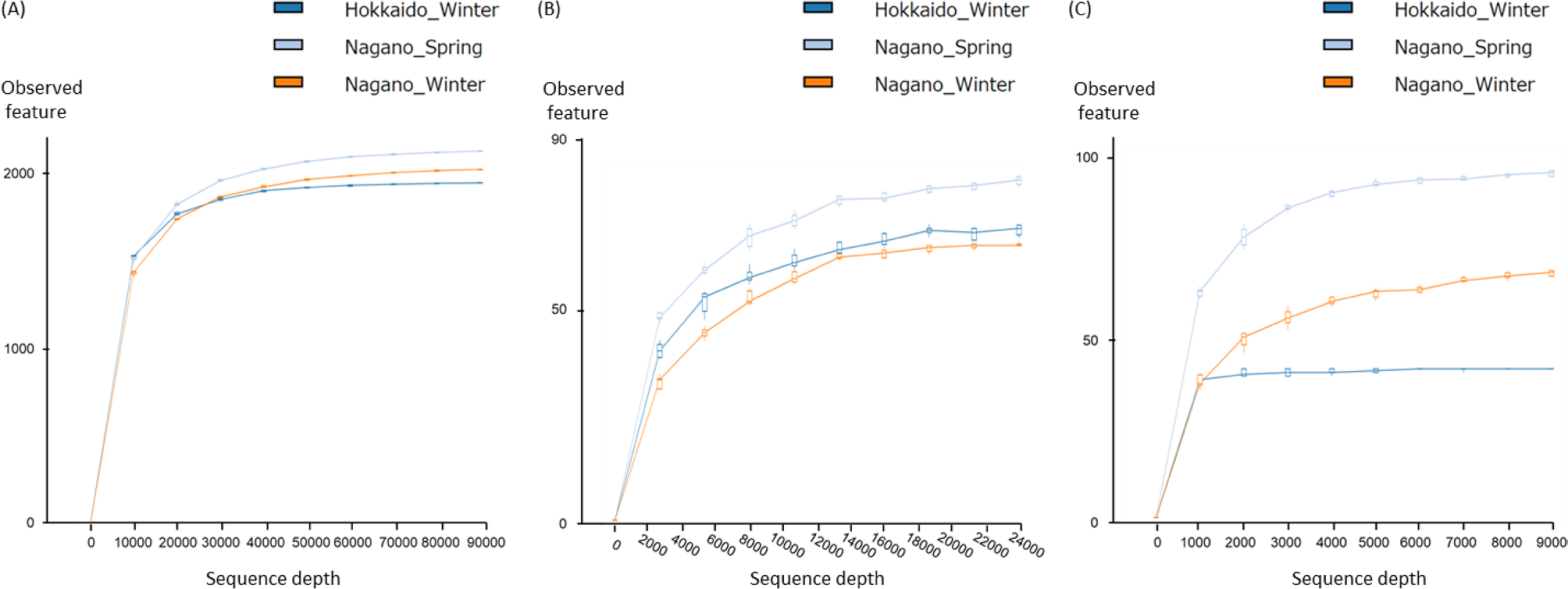
Alpha diversity of ruminal microbial and chloroplast composition in amplicon-seq analysis. Rarefaction curves of the observed OTUs are shown. (**A**) Bacterial taxa in the 16S rRNA dataset, (**B**) eukaryotic taxa without chloroplasts and host mitochondria in the 18S rRNA dataset, and (**C**) chloroplast taxa in the 18S rRNA dataset. Blue represents Hokkaido winter, pale blue represents Nagano spring, and orange represents Nagano winter.

The mean read count per sample, OTUs per sample, alpha diversity and related statistics for season and location groups (NW, NS, and HW) of the ruminal microbial community diversity including Bac, Euk, and Chl are summarized in Table 1. For all Bac, Euk, and Chl amplicon-seq data, rarefaction curves were constructed to ensure a sufficient sequencing depth (89,000, 24,000, and 9000 reads, respectively) for evaluating the dominant ruminal microbial and chloroplast composition (Fig. 1). In the Bac analysis, significant differences were not observed among the groups; however, in the Euk analysis, significant differences were observed among the groups in terms of the Shannon index (p < 0.01) and Pielou’s evenness (p < 0.01). In particular, the NW group showed the lowest eukaryotic richness (q < 0.05) and evenness (q < 0.05). In the Chl analysis, significant differences among the groups were observed only for observed OTUs (p < 0.05) and the Shannon index (p < 0.05) (Table 1).

**Table 1:** Number of read counts, OTUs, and results of alpha-diversity metrics in the ruminal microbial and chloroplast composition of sika deer summarized by season and location.

Ruminal community	Groups^a^	Number of samples	Read count/sample^b^	OTU/sample^b^	Observed OTUs^b^	q-value^c^	Shannon^b^	q-value^c^	Faith’s PD^b^	q-value^c^	Pielou’s evenness^b^	q-value^c^
Bacteria	NW	12	303,368 (104,305)	348.4 (48.4)	1873.8 (481.3)	0.83	8.9 (0.7)	0.41	126.2 (23.4)	0.54	0.82 (0.04)	0.10
	NS	10	256,181 (30,447)	388.5 (25.1)	2174.8 (322.7)	0.59	9.1 (0.6)	0.43	145.5 (14.2)	0.51	0.82 (0.04)	0.10
	HW	5	168,101 (10,281)	397.8 (12.4)	1998.4 (171.7)	0.59	9.5 (0.3)	0.41	141.4 (8.1)	0.23	0.86 (0.02)	0.90
	p-value^d^				0.42		0.34		0.18		0.12	
Eukaryota^e^	NW	12	302,958 (171,675)	44.9 (16.4)	65.4 (20.9)	0.71	1.4 (0.5)	0.02	14.6 (5.0)	0.75	0.24 (0.09)	0.02
	NS	10	191,784 (72,609)	48.9 (6.9)	78.0 (16.1)	0.49	2.4 (0.6)	0.002	17.4 (4.0)	0.21	0.38 (0.09)	0.006
	HW	5	166,911 (53,320)	44.8 (16.7)	71.4 (27.1)	0.49	2.2 (0.5)	0.54	13.3 (2.5)	0.21	0.36 (0.07)	0.62
	p-value^d^				0.37		0.001		0.17		0.003	
Chloroplast	NW	12	114,621 (98,009)	35.6 (11.7)	70.3 (28.9)	0.23	2.6 (0.8)	0.29	2.5 (0.4)	0.14	0.4 (0.1)	0.14
	NS	10	62,179 (26,875)	44.8 (8.4)	107.7 (36.4)	0.06	3.7 (0.8)	0.05	2.6 (0.4)	0.69	0.6 (0.1)	0.69
	HW	5	30,892 (23,085)	26.2 (9.2)	53.4 (24.7)	0.05	2.8 (0.8)	0.17	2.0 (0.4)	0.14	0.5 (0.1)	0.14
	p-value^d^				0.02		0.036		0.14		0.21	

^a^NW, Nagano winter; NS, Nagano spring; HW, Hokkaido winter.

^b^() indicates standard deviation.

^c^q-values for each pair were calculated by pairwise Kruskal–Wallis test. q-values in NW, NS, and HW show the statistics against HW, NW, and NS, respectively.

^d^p-values for all groups were calculated by Kruskal–Wallis test.

^e^Mitochondria and chloroplast taxa were excluded.

To examine the factors affecting ruminal microbial and chloroplast richness, a generalized linear model was applied to the OTUs identified in 27 sika deer based on Bac, Euk, and Chl sequences, including groups, sex, and age as factors. The group factor (NS) significantly predicted Chl diversity (p < 0.05). The analysis of deviance also showed significant differences only for groups in the Chl analysis (p < 0.05) and not for age and sex in the Bac, Euk, and Chl analyses (Supplementary Table S3).

### Differences in ruminal microbial and chloroplast composition among sampling seasons and locations revealed using amplicon-seq

**Figure 2:**
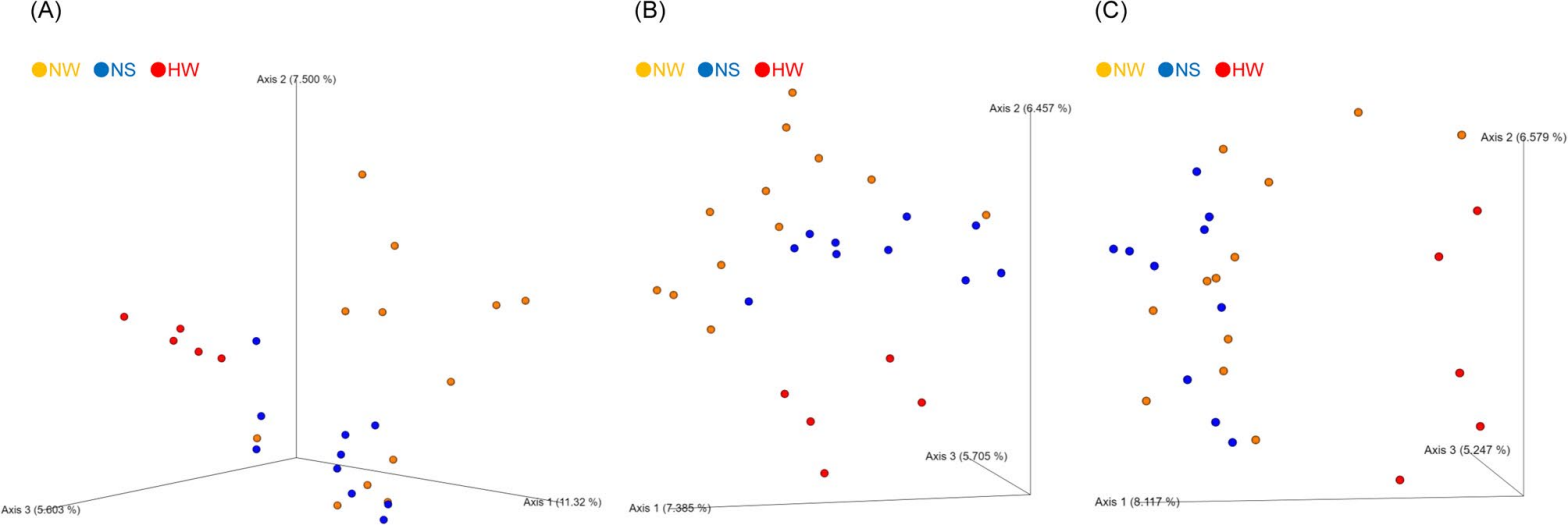
Beta diversity of ruminal microbial and chloroplast composition in amplicon-seq analysis. Beta diversity is shown using 3D plots of Jaccard distances. (**A**) Bacterial taxa in the 16S rRNA dataset, (**B**) eukaryotic taxa without chloroplasts and host mitochondria in the 18S rRNA dataset, and (**C**) chloroplast taxa in the 18S rRNA dataset. Circles indicate Hokkaido winter (HW) in red, Nagano spring (NS) in blue, and Nagano winter (NW) in orange.

**Figure 3:**
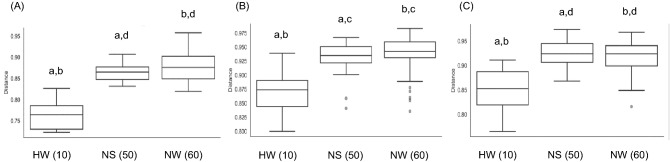
Beta diversity of ruminal microbial and chloroplast composition in amplicon-seq analysis. Comparisons among groups based on Jaccard distance metrics. (**A**) Bacterial taxa in the 16S rRNA dataset, (**B**) eukaryotic taxa without chloroplasts and host mitochondria in the 18S rRNA dataset, and (**C**) plant taxa in the 18S rRNA dataset. The distances from HW are shown. Differences in structure among groups were detected by PERMANOVA/ANOSIM. The same letters (a, b, c, and d) show statistically significant differences between groups (a, b, and c, p < 0.01; d, p < 0.05). (), number of distances calculated between samples within each group.

For the beta diversity metrics shown in Table 2, between NS and HW, statistically significant differences were observed in Jaccard (q < 0.01), Bray–Curtis (q < 0.01), and unweighted UniFrac (q < 0.01) distances for Bac, Jaccard (q < 0.01) and unweighted UniFrac (q < 0.05) distances for Euk, and Jaccard (q < 0.01) and Bray–Curtis (q < 0.01) distances for Chl. In particular, large differences (R > 0.7) in Jaccard and Bray–Curtis distances were observed for Bac. Differences in Jaccard (q < 0.01) and Bray–Curtis (q < 0.01) distances were observed between NW and HW for Bac, Jaccard (q < 0.01) distances for Euk, and Jaccard (q < 0.01), Bray–Curtis (q < 0.01), and weighted UniFrac (q < 0.05) distances for Chl. In particular, a large difference (R > 0.7) in Jaccard distances was observed for Bac. Differences between NS and NW were observed in Jaccard (q < 0.05) and Bray–Curtis (q < 0.05) distances for Bac, Jaccard (q < 0.01) distances for Euk, and Jaccard (q < 0.05) and Bray–Curtis (q < 0.05) distances for Chl. 3D PCoA plots of Jaccard distances are presented in Fig. 2. The compositions differed among the groups, as shown by the box and whisker plots generated from Jaccard distance metrics in Fig. 3. From most to least diverse, the groups were ordered NW, NS, and HW for Bac and Euk and NS, NW, and HW for Chl.

**Table 2:** Statistical results of beta diversity metrics in the ruminal bacterial and chloroplast composition summarized by season and location.

Groups^a^	Statistics^b^	Ruminal community											
		Bacteria				Eukaryota^c^				Chloroplast			
		Jaccard	Bray–Curtis	Unweighted UniFrac	Weighted UniFrac	Jaccard	Bray–Curtis	Unweighted UniFrac	Weighted UniFrac	Jaccard	Bray–Curtis	Unweighted UniFrac	Weighted UniFrac
All (27)	R	0.50	0.45	0.25	0.047	0.36	0.10	0.12	0.10	0.29	0.29	0.095	0.15
	p-value	0.001	0.001	0.003	0.21	0.001	0.093	0.037	0.076	0.001	0.001	0.092	0.026
NS vs HW (15)	R	1	0.91	0.72	0.12	0.50	0.34	0.30	0.21	0.66	0.47	0.16	0.11
	q-value	0.0045	0.003	0.006	0.17	0.003	0.06	0.03	0.14	0.003	0.002	0.19	0.16
NW vs HW (17)	R	0.79	0.67	0.26	−0.077	0.39	0.34	0.15	0.055	0.33	0.42	−0.012	0.34
	q-value	0.0045	0.003	0.05	0.65	0.003	0.21	0.20	0.23	0.008	0.002	0.48	0.039
NS vs NW (22)	R	0.15	0.14	0.11	0.075	0.28	0.34	0.04	0.10	0.11	0.13	0.12	0.058
	q-value	0.013	0.021	0.05	0.24	0.003	0.32	0.25	0.14	0.033	0.041	0.11	0.16

^a^() Number of samples. NW, Nagano winter; NS, Nagano spring; HW, Hokkaido winter.

^b^ANOSIM, Number of permutations = 999.

^c^Mitochondria and chloroplast taxa were excluded.

Only the samples from adult male deer showed tendencies toward significant differences in Jaccard distances among the groups (Supplementary Fig. S1). There was no significant difference in the Jaccard distances between each age category (adult, subadult and young) and sex (female and male) within the same group (HW, NS and NW) (Supplementary Figs. S2 and S3).

### Taxonomic analysis of ruminal microbial and chloroplast composition using amplicon-seq

**Figure 4:**
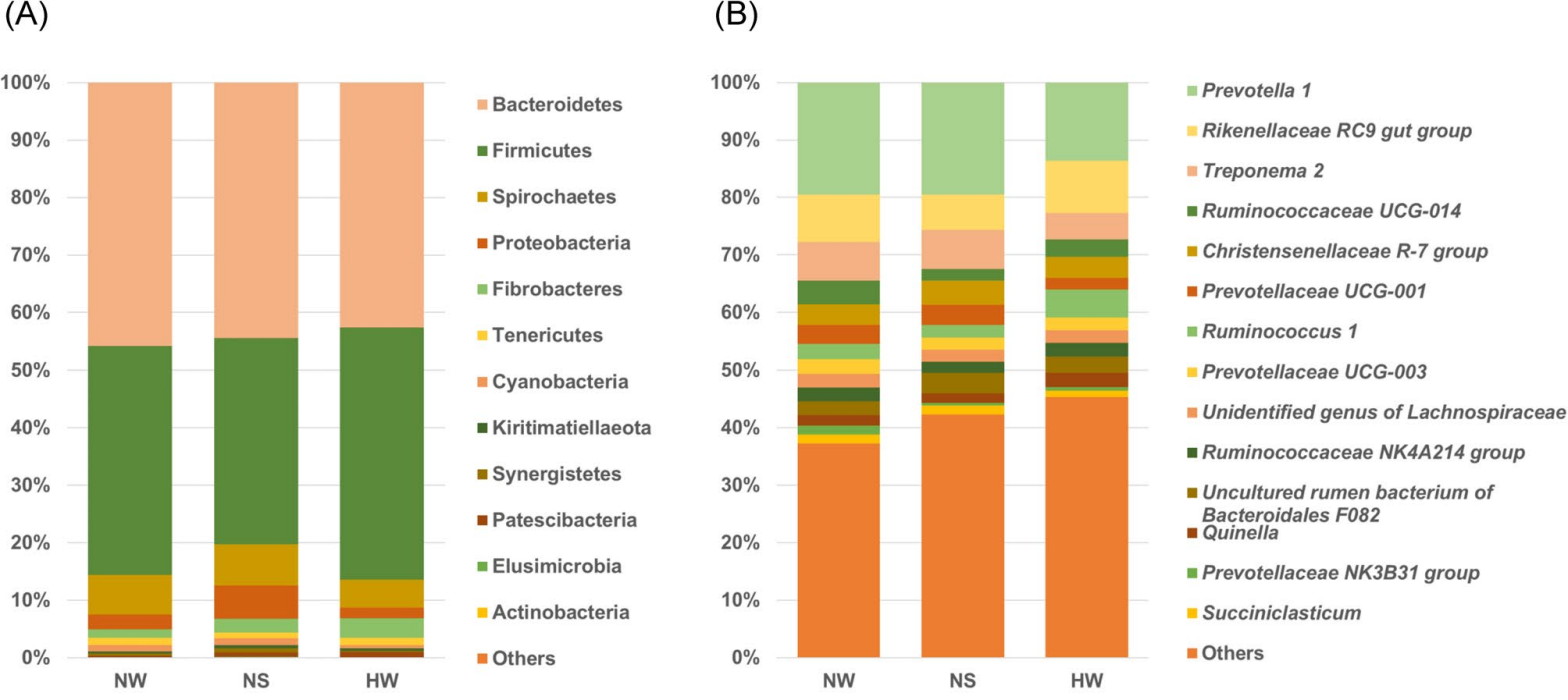
Average relative abundance of bacterial taxa at the phylum (**A**) and genus (**B**) levels in the rumen bacterial composition of each group. *NW* Nagano winter, *NS* Nagano spring, *HW* Hokkaido winter. The bacterial taxa were obtained from 16S rRNA amplicon-seq analysis.

The mean OTUs at the phylum and genus levels in the groups are described in Supplementary Tables S4 to S9. The top three Bac phyla (mean % of NW, NS, and HW) were Bacteroidetes (45.4, 44.2, and 42.0%), Firmicutes (39.5, 35.6, and 43.4%), and Spirochaetes (6.8, 7.1, and 4.8%) (Fig. 4A, Supplementary Table S4). The top Bac genera (mean %) were *Prevotella* 1 (19.5, 19.5, and 13.6%), *Rikenellaceae* RC9 gut group (8.2, 6.1, and 9.1%), and *Treponema* 2 (6.7, 6.8, and 4.6%). (Fig. 4B, Supplementary Table S1).

**Figure 5:**
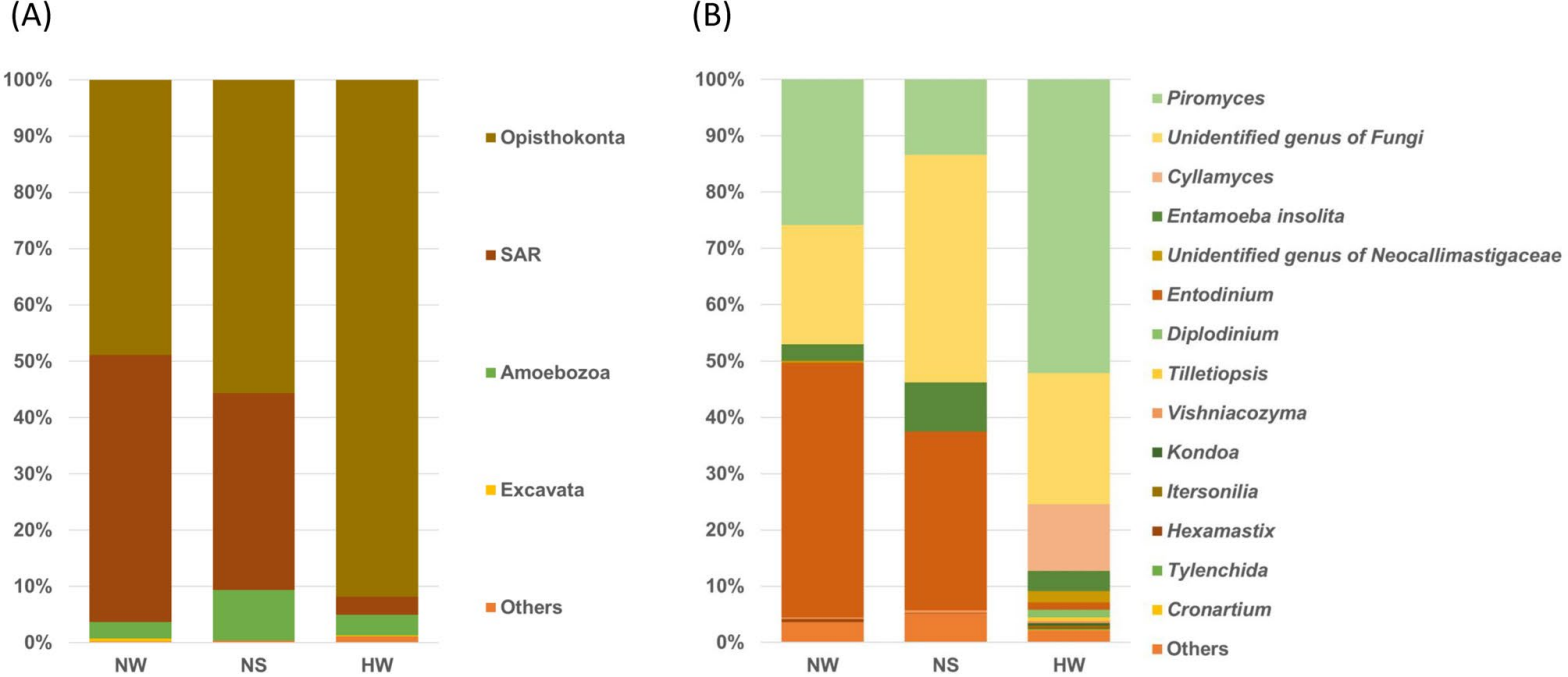
Average relative abundance of eukaryotic taxa at the phylum (**A**) and genus (**B**) levels in the rumen eukaryotic composition of each group. *NW* Nagano winter, *NS* Nagano spring, *HW* Hokkaido winter. Eukaryotic taxa excluded chloroplasts and host mitochondria from 18S rRNA amplicon-seq analysis.

The top three Euk phyla (the mean %) were Opisthokonta (48.9, 55.8, and 91.8%), SAR (an acronym of Stramenopiles, Alveolata, and Rhizaria) (47.4, 35.0, and 3.2%), and Amoebozoa (2.9, 9.0, and 3.7%) (Fig. 5A, Supplementary Table S6). The top Euk genera were *Entodinium* (45.2, 31.7, and 1.3%), *Piromyces* (25.8, 13.3, and 51.9%), and unidentified fungal taxa (21.1, 40.3, and 23.3%). *Cyllamyces* predominated only in HW (11.7%) (Fig. 5B, Supplementary Table S7).

**Figure 6:**
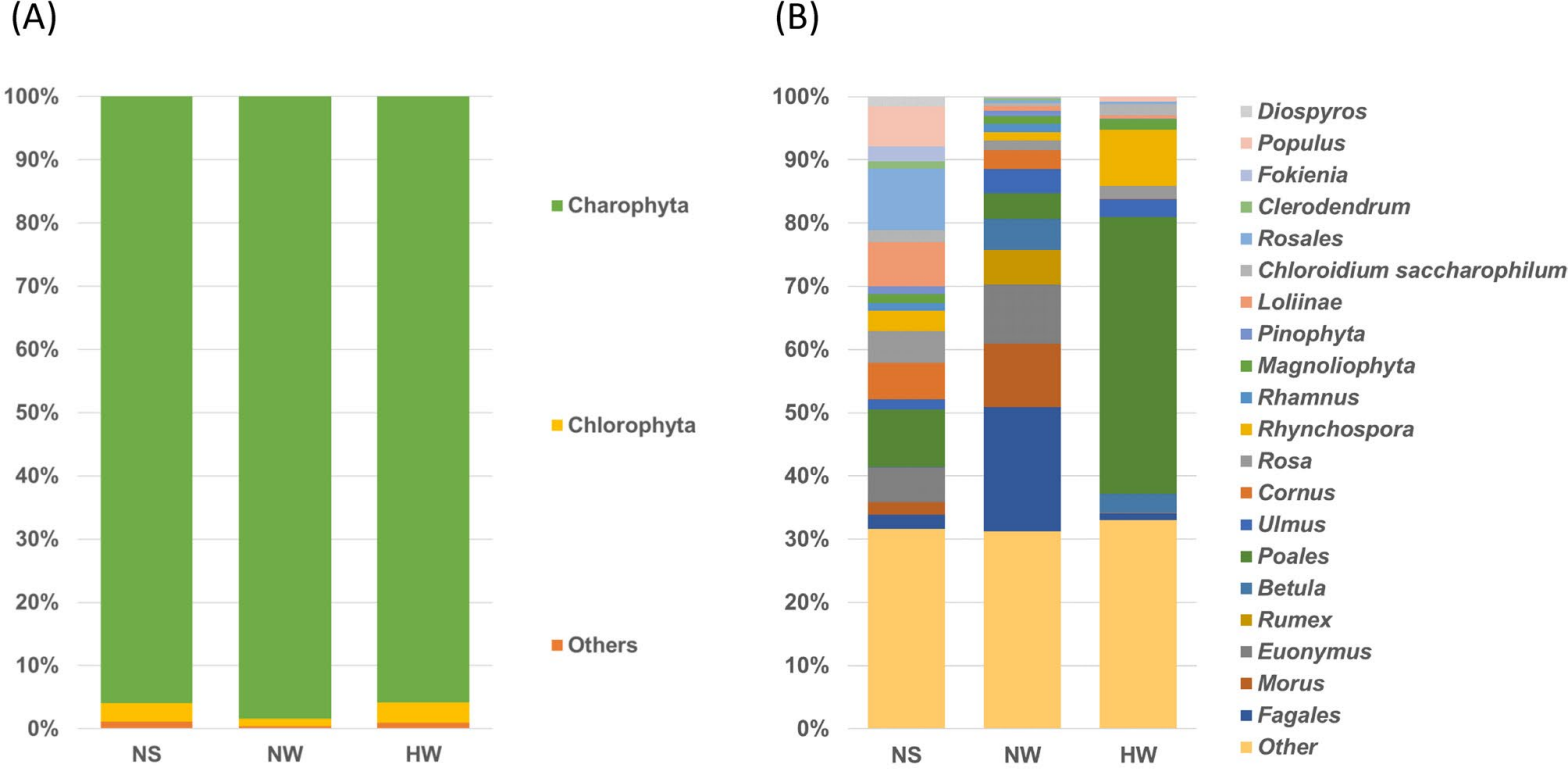
Average relative abundance of chloroplast taxa at the phylum (**A**) and genus (**B**) levels in the rumen chloroplast composition of each group. *NW* Nagano winter, *NS* Nagano spring, *HW* Hokkaido winter. The plant taxa were obtained from 18S rRNA amplicon-seq analysis.

Over 90% of the Chl phyla were members of Charophyta (the mean%) (98.4, 95.9, and 95.8%) (Fig. 6A, Supplementary Table S8). The top Chl genera were unidentified Fagales taxa (19.7%) in NW, unidentified Charophyta taxa (21.5%) in NS, and unidentified Poales taxa (43.8%) in HW. The second most common were unidentified Charophyta taxa in NW (15.6%) and HW (17.8%) and unidentified Rosales taxa (9.7%) in NS. The third most abundant taxa were *Morus* (10.1%) in NW, unidentified Poales taxa (9.2%) in NS, and *Rhynchospora* (8.9%) in HW (Fig. 6B, Supplementary Table S9).

### Taxon-level differences in ruminal microbial and chloroplast composition detecting using amplicon-seq

**Figure 7:**
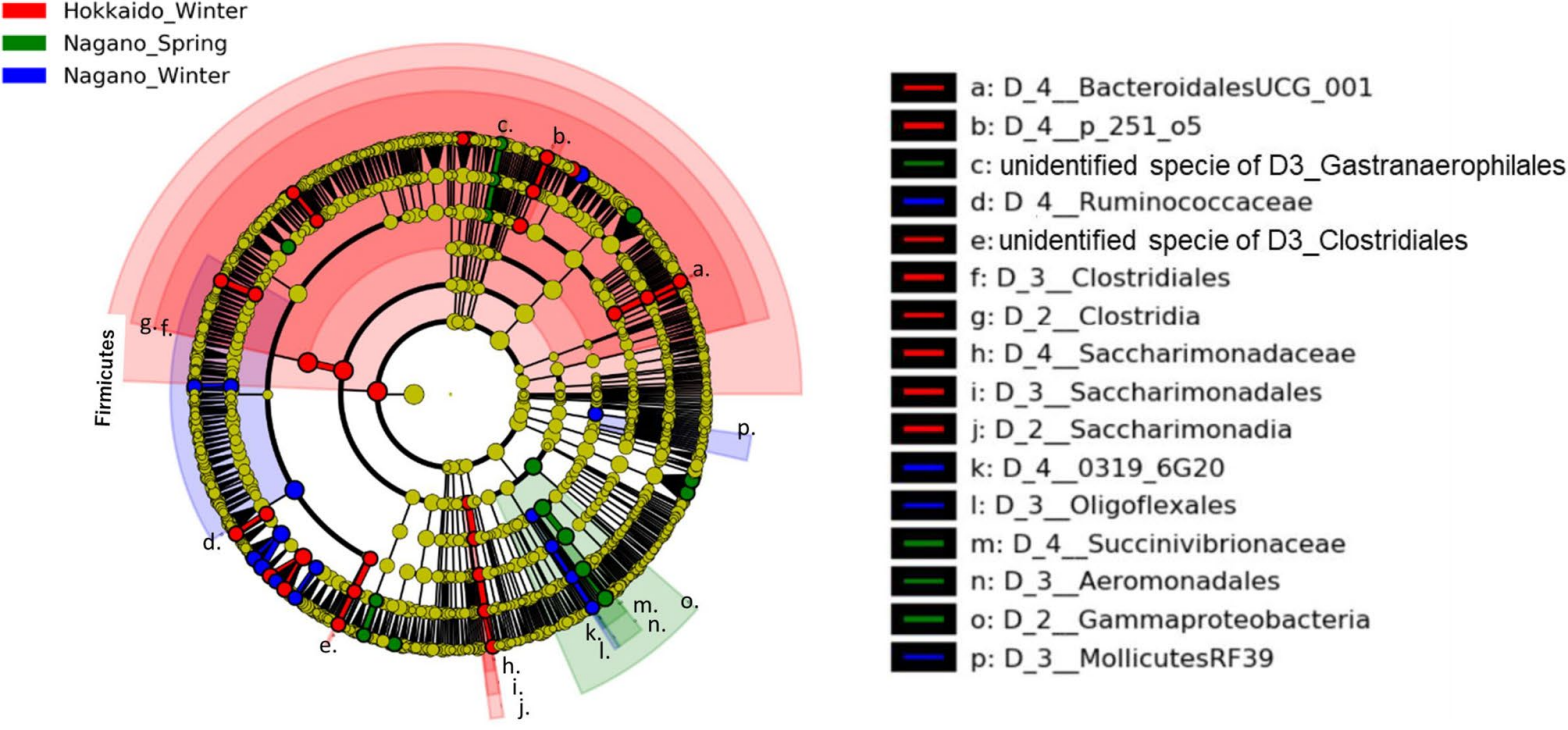
Cladograms of bacterial taxa in ruminal bacterial composition detected by the LEfSe approach. Red, green, and blue indicate significantly different groups (Hokkaido winter, Nagano spring, and Nagano winter, respectively), with the diagram and species classification at the phylum, class, order, family, and genus levels shown from the inside to the outside. Yellow circles represent species with no significant difference. Letters in the cladograms indicate the significantly different taxa in each clade including more than 2 taxa.

**Figure 8:**
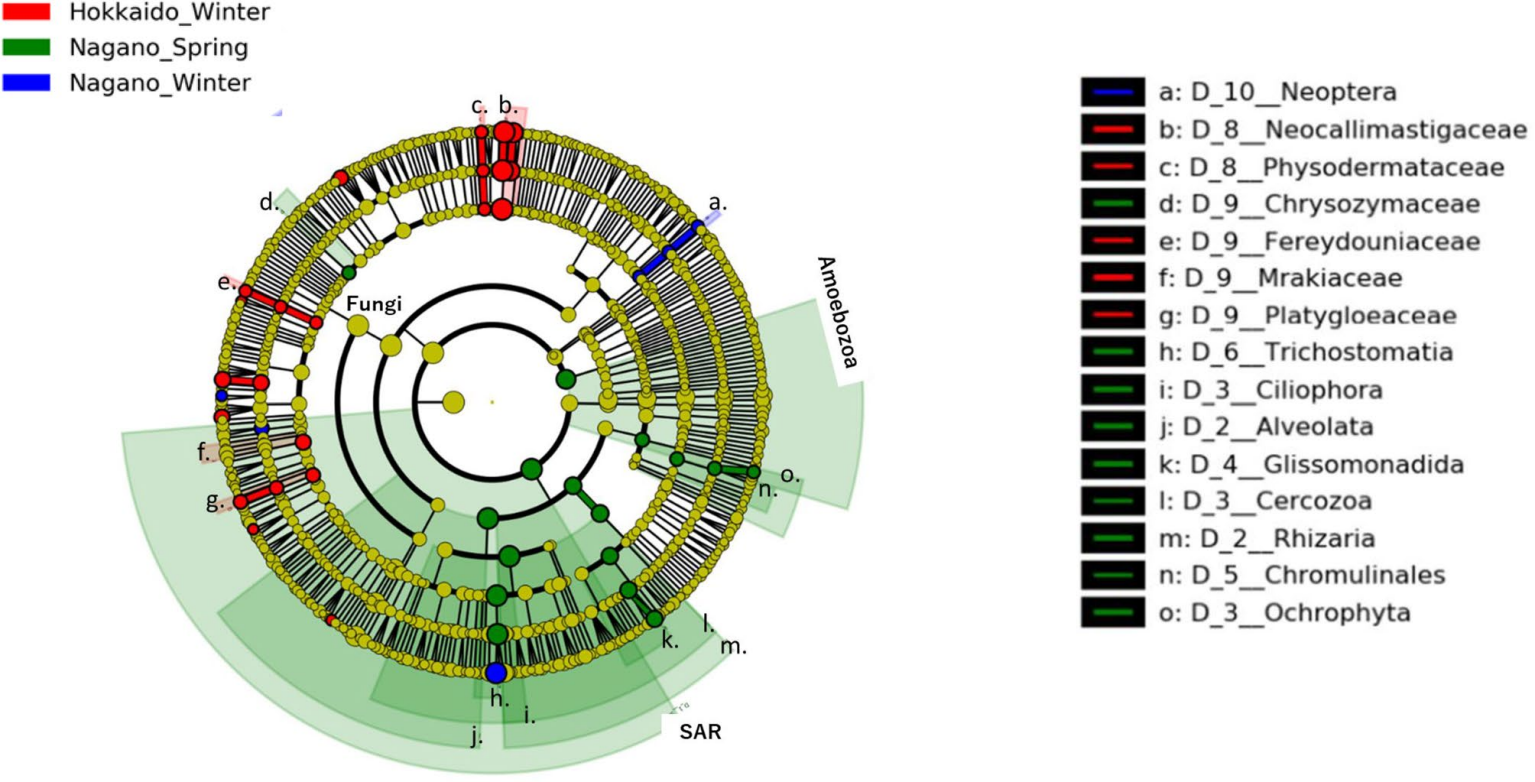
Cladograms of eukaryotic taxa in ruminal eukaryal composition detected by the LEfSe approach. Red, green, and blue indicate significantly different groups (Hokkaido winter, Nagano spring, and Nagano winter, respectively), with the diagram and species classification at the phylum, class, order, family, and genus levels shown from the inside to the outside. Yellow circles represent species with no significant difference. Letters in the cladograms indicate the significantly different taxa in each clade including more than 2 taxa. Eukaryotic taxa excluded chloroplasts and host mitochondria from 18S rRNA amplicon-seq analysis.

**Figure 9:**
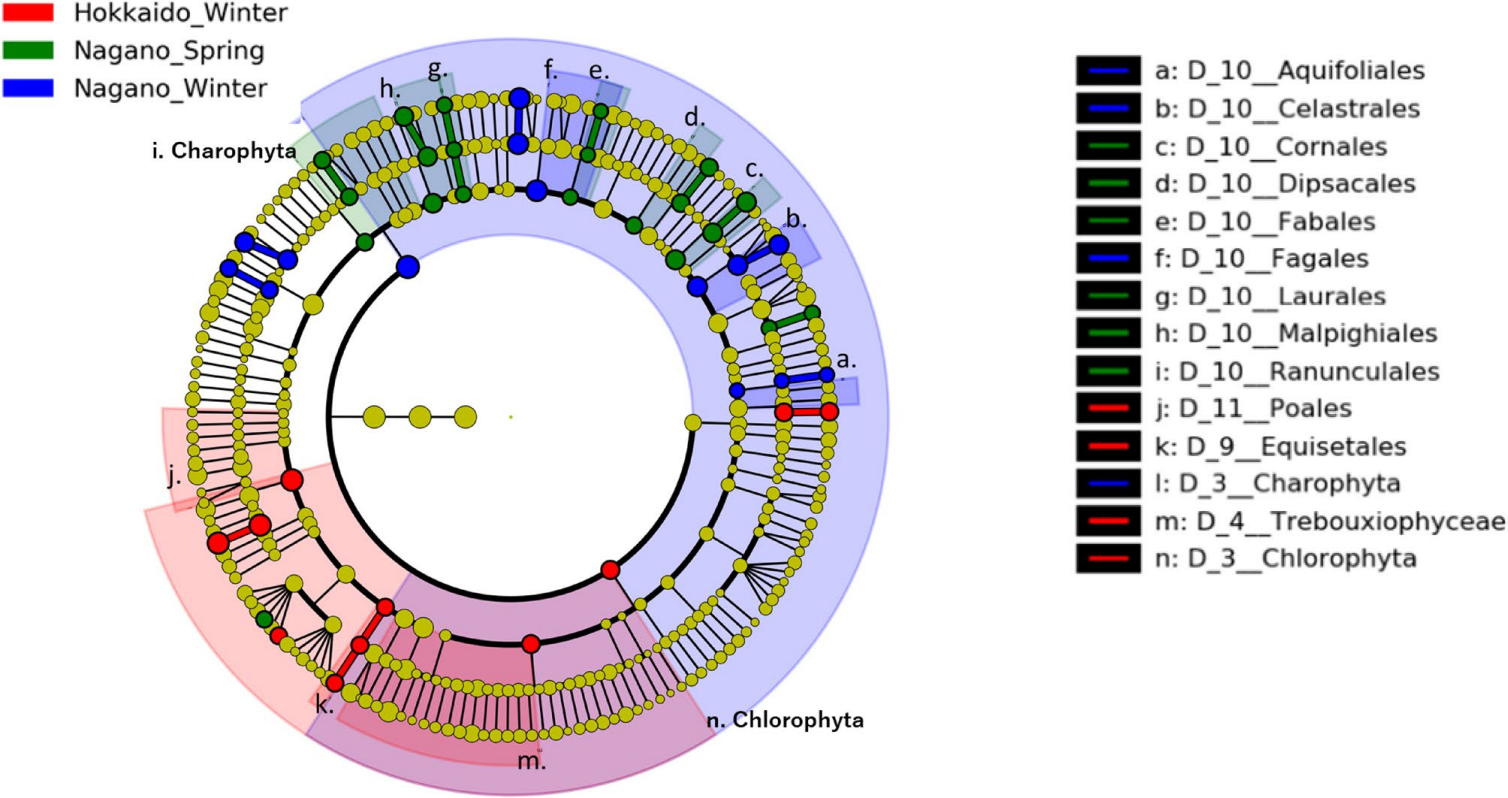
Cladograms of chloroplast taxa in ruminal chloroplast composition detected by the LEfSe approach. Red, green, and blue indicate significantly different groups (Hokkaido winter, Nagano spring, and Nagano winter, respectively), with the diagram and species classification at the phylum, class, order, family, and genus levels shown from the inside to the outside. Yellow circles represent species with no significant difference. Letters in the cladograms indicate the significantly different taxa in each clade including more than 2 taxa. The plant taxa were obtained from 18S rRNA amplicon-seq analysis.

The linear discriminant analysis (LDA) effect size (LEfSe) technique was used to compare taxon abundances among sampling seasons and locations at the species level (level 7). The LEfSe technique identified 66 Bac taxa, 44 Euk taxa, and 46 Chl taxa with LDA scores above 3 and significant differences (p < 0.05) among NS, NW, and HW (Supplementary Tables S10 to S12). Relevant features of these taxonomic or phylogenetic trees were visualized by cladograms (Figs. 7, 8, 9).

Of the 66 Bac taxa, 27 showed a significant difference (p < 0.01) among groups (Fig. 7 and Supplementary Table S10). Of the 27 Bac taxa, the taxa identified in HW were g_*Lachnospiraceae* XPB1014 group, g_*Lachnoclostridium* 10, *g_Ruminococcus* 1, s_*Fibrobacter* UWB11, s_*F. aceaebacterium*, and s_*F. succinogenes* subsp. *succinogenes*, whereas those in NS were g_*Ruminobacter* and s_*Treponema* AC3 and those in NW were f_*Ruminococcaceae*, f_*Oligoflexales* 0319 6G20, and s_*Ruminococcaceae* UCG_014.

Of the 44 Euk taxa, 9 showed a significant difference (p < 0.01) among groups (Fig. 8 and Supplementary Table S11). Of the 9 Euk taxa, the taxon identified in HW was f_Neocallimastigaceae, whereas the taxa identified in NS belonged to the infrakingdom Rhizaria, p_Cercozoa, p_Ochrophyta, o_Glissomonadida, o_Chromulinales, g_*Spumella*, and g_*Heteromita*.

Of the 46 Chl taxa, 13 showed a significant difference (p < 0.01) among groups (Fig. 9 and Supplementary Table S12). Of the 13 Chl taxa, the taxa identified in HW were o_Poales and g_*Panax*, whereas those in NS were o_Fabales, o_Laurales, g_*Cinnamomum*, and *C. camphora*.

### Differential expression in ruminal microbes detected using total RNA-seq analysis

A total of 2998 predicted open reading frame (ORF) sequences (predicted transcripts) were obtained from a total of 4,356,106 read counts (mean 161,337 ± 41,251 SD reads per sample). The normalization and differential expression analysis of the predicted transcripts were performed using TCC-baySeq. Of the 2998 predicted transcripts, 397 statistically significant differentially expressed (DE) transcripts (q < 0.05) were obtained by clearing 56 non-DE transcripts. Of the 397 DE transcripts, the numbers for each comparison (HW > others, NS > others, NW > others, others > HW, others > NS, and others > NW) were 193, 70, 18, 53, 38, and 25, respectively. The results of the homology search using BLAST + against the Swiss-Prot database showed the annotation of 119 DE transcripts among the 397 DE transcripts. The predicted taxa for the DE transcripts annotated by Swiss-Prot for each comparison are summarized in Table 3, and the raw numerical data for the DE analysis are provided in Supplementary Table S13.

**Table 3:** Group-specific predicted taxa in the ruminal microbes annotated by Swiss-Prot and analyzed by TCC-baySeq (q-value < 0.05).

Order	Estimate taxon annotated for the estimate transcripts using Swiss-Prot	Description in Swiss-Prot (Number of annotations)
HW > other	*Bacteria; Firmicutes; Clostridia; Clostridiales; Clostridiaceae; Clostridium*	Glyceraldehyde-3-phosphate dehydrogenase (9), Flagellin (4)
	*Bacteria; Bacteroidetes; Bacteroidia; Bacteroidales; Bacteroidaceae; Bacteroides*	Enolase (8)
	*Bacteria; Firmicutes; Bacilli; Bacillales; Bacillaceae; Bacillus*	Flagellin (6), Phosphocarrier protein HPr (1)
	*Bacteria; Firmicutes; Bacilli; Bacillales; Staphylococcaceae; Staphylococcus*	Phosphoenolpyruvate-protein phosphotransferase (2), Phosphocarrier protein HPr (2)
	*Bacteria; Firmicutes; Negativicutes; Selenomonadales; Selenomonadaceae; Selenomonas*	l-lactate dehydrogenase (4)
	*Bacteria; Spirochaetes; Spirochaetales; Spirochaetaceae; Treponema*	Flagellin FlaB1 (3), Fructose-bisphosphate aldolase (3), Flagellar filament 33 kDa core protein (1)
	*Bacteria; Firmicutes; Bacilli; Bacillales; Bacillaceae; Geobacillus*	Glycogen biosynthesis protein GlgD (2), Glucose-1-phosphate adenylyltransferase (1)
	*Bacteria; Firmicutes; Clostridia; Thermoanaerobacterales; Thermoanaerobacteraceae; Carboxydothermus*	Glucose-6-phosphate isomerase (2), Phosphoglycerate kinase (1)
	*Bacteria; Proteobacteria; Gammaproteobacteria; Enterobacterales; Enterobacteriaceae; Escherichia*	Raffinose invertase (2)
	*Bacteria; Proteobacteria; Gammaproteobacteria; Legionellales; Legionellaceae; Tatlockia*	Flagellin (2)
	*Bacteria; Firmicutes; Bacilli; Lactobacillales; Lactobacillaceae; Lactobacillus*	Phosphoenolpyruvate-protein phosphotransferase (1)
	*Bacteria; Firmicutes; Bacilli; Lactobacillales; Streptococcaceae; Streptococcus*	Glyceraldehyde-3-phosphate dehydrogenase (1)
	*Bacteria; Proteobacteria; Alphaproteobacteria; Sphingomonadales; Sphingomonadaceae; Zymomonas*	Sucrose-6-phosphate hydrolase (1)
	*Bacteria; Proteobacteria; Betaproteobacteria; Neisseriales; Neisseriaceae; Neisseria*	Iron-regulated protein FrpC (1)
	*Bacteria; Proteobacteria; Deltaproteobacteria; Desulfuromonadales ; Geobacteraceae; Geobacter*	4-hydroxy-3-methylbut-2-enyl diphosphate reductase (1)
	*Bacteria; Proteobacteria; Gammaproteobacteria; Enterobacterales; Pectobacteriaceae; Dickeya*	Serralysin C (1)
	*Bacteria; Spirochaetes; Brachyspirales; Brachyspiraceae; Brachyspira*	Flagellar filament core protein flaB2 (1)
	*Eukaryota; Viridiplantae; Streptophyta; Embryophyta; Tracheophyta; Spermatophyta; Magnoliopsida; Liliopsida; Asparagales; Orchidaceae; Epidendroideae; Vandeae; Aeridinae; Phalaenopsis*	Uncharacterized protein ORF91 (2)
	*Eukaryota; Metazoa; Chordata; Craniata; Vertebrata; Euteleostomi; Mammalia; Eutheria; Laurasiatheria; Perissodactyla; Equidae; Equus*	1,4-alpha-glucan-branching enzyme (1)
	*Eukaryota; Metazoa; Ecdysozoa; Arthropoda; Crustacea; Multicrustacea; Malacostraca; Eumalacostraca; Eucarida; Decapoda; Pleocyemata; Achelata; Palinuroidea; Palinuridae; Panulirus*	Glyceraldehyde-3-phosphate dehydrogenase (1)
NS > other	*Viruses; Riboviria; Solemoviridae; Sobemovirus*	Movement protein P1 (2), Polyprotein P2A (6), Replicase polyprotein P2AB (4), Capsid protein (2)
	*Viruses; Riboviria; Tombusviridae; Procedovirinae; Betanecrovirus*	RNA-directed RNA polymerase (9)
	*Viruses; Riboviria; Tombusviridae; Procedovirinae; Alphanecrovirus*	Capsid protein (8)
	*Viruses; Riboviria; Orthornavirae; Kitrinoviricota; Alsuviricetes; Martellivirales; Bromoviridae; Cucumovirus Hemiptera*	Capsid protein (1)
NW > other	*Eukaryota; Fungi; Dikarya; Ascomycota; Taphrinomycotina; Schizosaccharomycetes; Schizosaccharomycetales; Schizosaccharomycetaceae; Schizosaccharomyces*	Heat shock protein 16 (1)
	*Eukaryota; Metazoa; Chordata; Craniata; Vertebrata; Euteleostomi; Amphibia; Batrachia; Anura; Pipoidea; Pipidae; Xenopodinae; Xenopus; Xenopus*	Polyubiquitin (1)
	*Viruses; Riboviria; Orthornavirae; Kitrinoviricota; Alsuviricetes; Martellivirales; Bromoviridae; Bromovirus*	RNA-directed RNA polymerase 2a (1), Replication protein 1a (1), Capsid protein (1), Movement protein (1)
	*Viruses; Riboviria; Tymovirales; Alphaflexiviridae; Potexvirus*	Movement and silencing protein TGBp1 (1)
Other > HW	*Bacteria; Firmicutes; Bacilli; Bacillales; Bacillaceae; Bacillus*	*N*-acetylmuramoyl-l-alanine amidase XlyA (1)
	*Eukaryota; Fungi; Dikarya; Ascomycota; Taphrinomycotina; Schizosaccharomycetes; Schizosaccharomycetales; Schizosaccharomycetaceae; Schizosaccharomyces*	Heat shock protein 16 (3)
	*Eukaryota; Viridiplantae; Streptophyta; Embryophyta; Tracheophyta; Spermatophyta; Magnoliopsida; Liliopsida; Poales; Poaceae; BOP clade; Oryzoideae; Oryzeae; Oryzinae; Oryza; Oryza sativa*	17.7 kDa class I heat shock protein (3)
	*Eukaryota; Fungi; Dikarya; Ascomycota; Pezizomycotina; Sordariomycetes; Hypocreomycetidae; Hypocreales; Hypocreaceae; Trichoderma*	14-3-3 protein homolog (1)
	*Eukaryota;* SAR*; Alveolata; Apicomplexa; Aconoidasida; Haemosporida; Plasmodiidae; Plasmodium; Plasmodium (Laverania)*	Elongation factor 1-alpha (1)
	*Eukaryota; Viridiplantae; Streptophyta; Embryophyta; Tracheophyta; Spermatophyta; Magnoliopsida; eudicotyledons; Gunneridae; Pentapetalae; rosids; malvids; Brassicales; Brassicaceae; Camelineae; Arabidopsis*	17.6 kDa class I heat shock protein 3 (1)
Other > NW	*Bacteria; Chlorobi; Chlorobia; Chlorobiales; Chlorobiaceae; Prosthecochloris*	Phosphoglycerate kinase (1)
	*Bacteria; Firmicutes; Clostridia; Clostridiales; Clostridiaceae; Clostridium*	Flagellin (1)
	*Bacteria; Proteobacteria; Gammaproteobacteria; Enterobacterales; Enterobacteriaceae; Klebsiella*	PTS system sucrose-specific EIIBC component (1)
Other > NS	*Eukaryota; Viridiplantae; Streptophyta; Embryophyta; Tracheophyta; Spermatophyta; Magnoliopsida; Liliopsida; Asparagales; Orchidaceae; Epidendroideae; Vandeae; Aeridinae; Phalaenopsis*	Uncharacterized protein ORF91 (2)
	*Eukaryota; Fungi; Dikarya; Ascomycota; Saccharomycotina; Saccharomycetes; Saccharomycetales; Saccharomycetaceae; Saccharomyces*	Regulator of rDNA transcription protein 15 (1)

### Correlation between the results of 16S rRNA amplicon-seq and total RNA-seq analysis

To use the results of amplicon-seq for the association analysis with total RNA-seq data, TCC-baySeq was applied to normalize the 16S rRNA amplicon-seq results at the species level (level 7). TCC-baySeq identified 35 different taxa (taxa with differential expression) (q < 0.01). Of 119 annotated DE transcripts in the total RNA-seq dataset, 48 annotated bacterial DE transcripts were obtained by clearing annotated eukaryote, fungus, and virus transcripts with a significance of q < 0.01.

**Figure 10:**
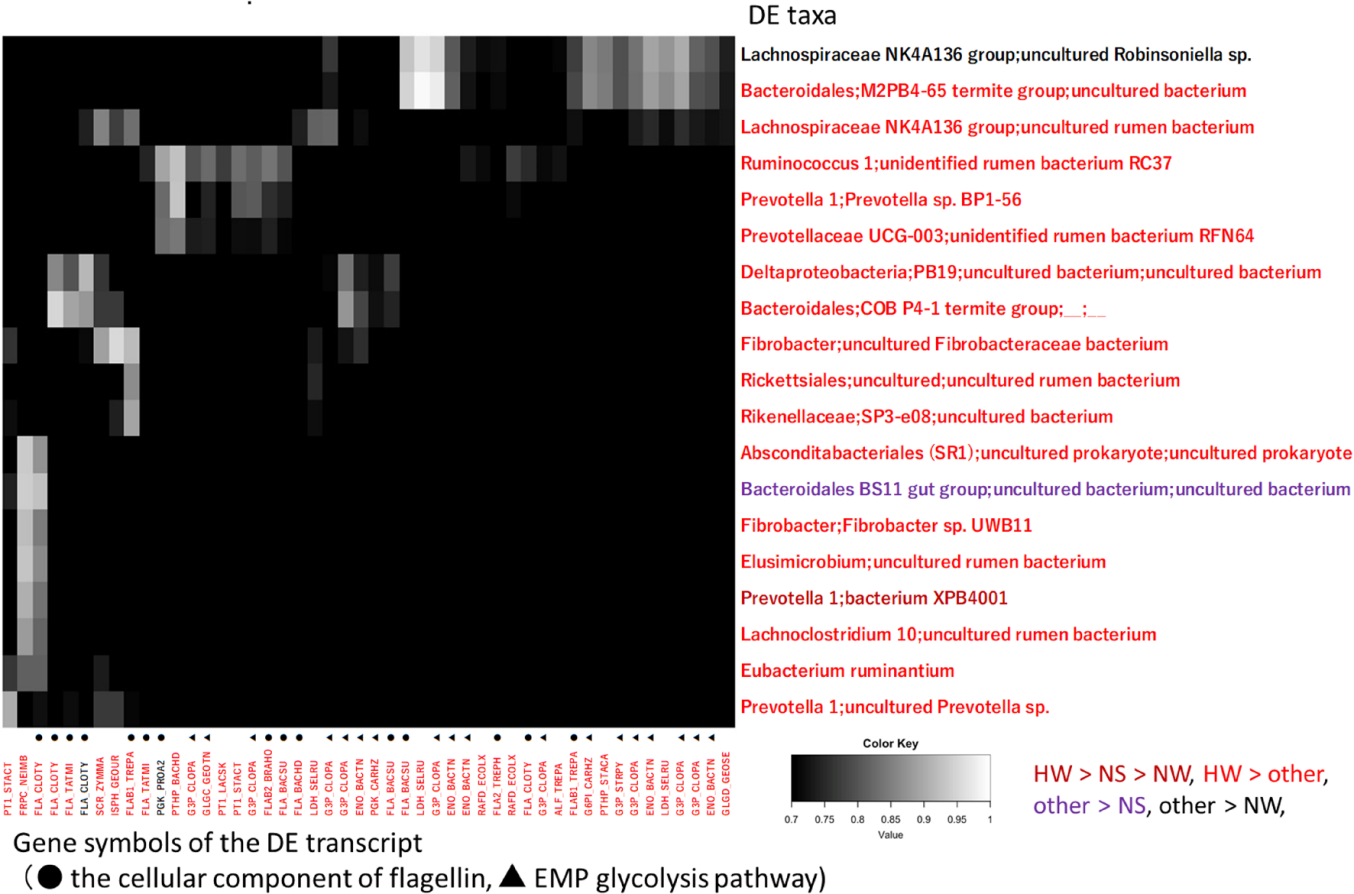
Pearson correlation coefficients of rumen microbial composition between amplicon-seq and total RNA-seq data. The taxonomy_7_levels.txt file in the SILVA database was used for annotation of bacterial OTUs in the amplicon-seq data. BLAST + against the Swiss-Prot database was used for the annotation of expected ORF sequences in the total RNA-seq data. Correlations with coefficients between 0.7 and 1.0 are shown. The color of the taxa with differential expression and gene symbols indicate statistically significant differences among the groups (q < 0.01) analyzed by TCC baySeq. The flagellin cellular component and EMP glycolysis pathway are shown as filled circles and triangles, respectively.

A total of 19 taxa with differential expression and 48 DE transcripts were detected to have at least 1 correlation and covariance of more than 0.7 (Fig. 10 and Supplementary Table S14). Almost all taxa with differential expression and DE transcripts were in a comparison with HW ordered first (17 and 46, respectively) when grouped by TCC-baySeq. The abundant taxa with differential expression that correlated with the DE transcripts (number) were as follows: g_*Prevotella* 1 (3), g_*Fibrobacter* (2), and g_*Lachnospiraceae* NK4A136 group (2). The abundant functions and pathways (gene symbols) of the DE transcripts correlated with the taxa showing differential expression were as follows: the cellular component of flagellin (FLA, FLA2, FLAB1, and FLAB2), the Embden–Meyerhof–Parnas (EMP) glycolysis pathway (ENO, G3P, GLGC, G6PI, and PGK), pyruvate fermentation to lactate (LDH), and phosphoenolpyruvate:carbohydrate phosphotransferase (PTS) (PT1 and PTHP).

### Correlation between the results of 16S rRNA amplicon-seq and PICRUSt2 analysis

The metagenomic functions of Bac taxa were predicted using PICRUSt2. A total of 383 pathways (mean 324 ± 17 SD pathways per sample) related to the MetaCyc pathway were identified in PICRUSt2 through structured mappings of Enzyme Commission (EC) gene families to pathways. The raw MetaCyc pathway data were used for the association analysis because the abundances had been normalized by the PICRUSt2 pipeline.

**Figure 11:**
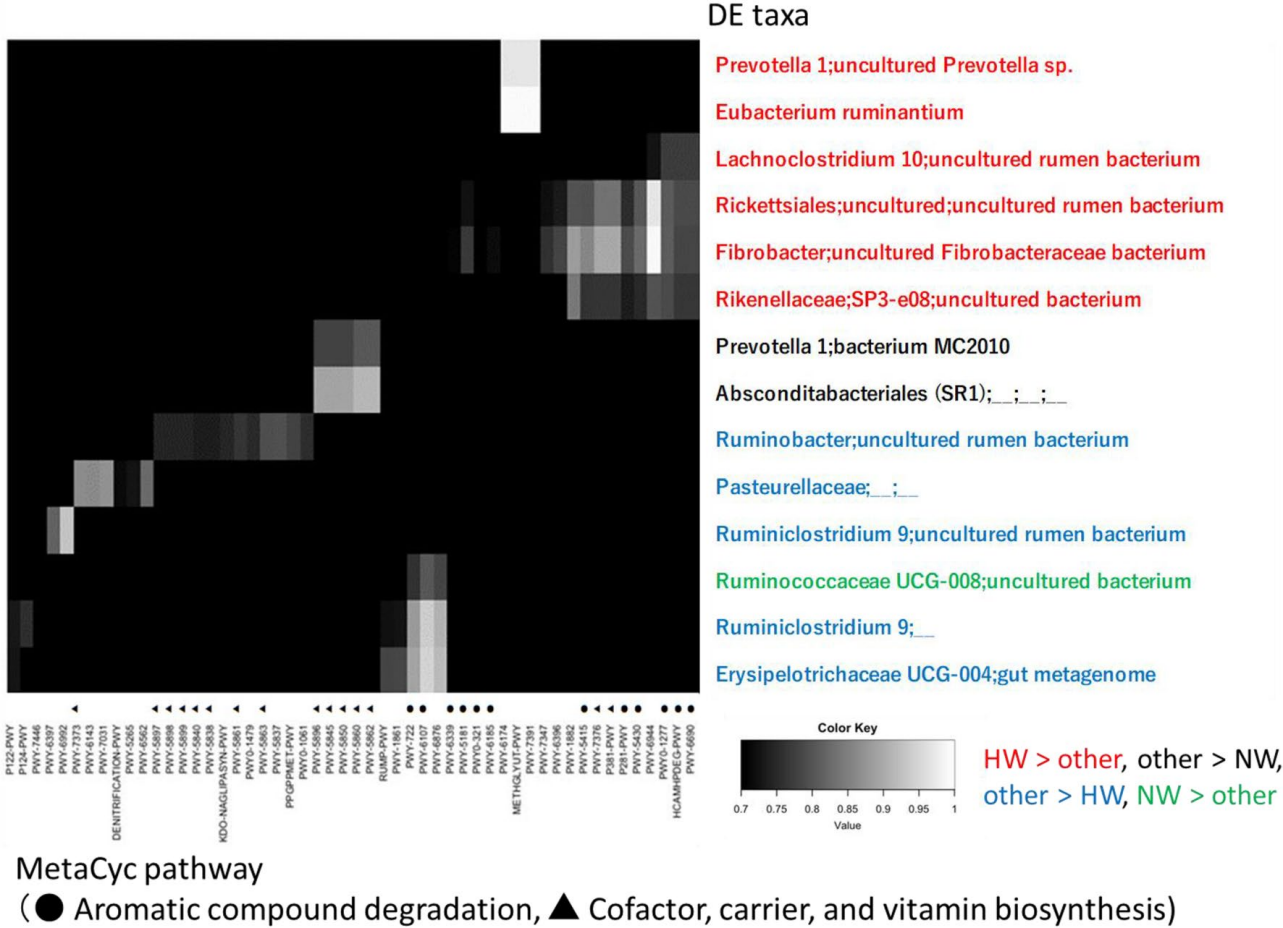
Pearson correlation coefficients between the results of 16S rRNA amplicon-seq and PICRUSt2 in the ruminal bacterial composition. The taxonomy_7_levels.txt file in the SILVA database was used for annotation of bacterial OTUs in the amplicon-seq data. The color of the DE taxa indicates a statistically significant difference among the groups (q < 0.01) analyzed by TCC baySeq. PICRUSt2 analysis was performed with dada2 output in QIIME2. The MetaCyc pathway was searched in the PICRUSt2 pipeline. Correlations with a coefficient between 0.7 and 1.0 are shown. Aromatic compound degradation and cofactor, carrier, and vitamin biosynthesis are shown as filled circles and triangles, respectively.

A total of 96 correlations (14 taxa with differential expression and 52 MetaCyc pathways) were detected to have at least 1 correlation and covariance of more than 0.7 (Fig. 11 and Supplementary Table S15). Of the 14 taxa with differential expression, the order predicted by TCC-baySeq was HW > other (number = 6 taxa), NW > other (1 taxon), other > HW (5 taxa), and other > NW (2 taxa). Of the 96 correlations (the number of each MetaCyc class of degradation/utilization/assimilation, biosynthesis, generation of precursor metabolites and energy, and macromolecule modification), 49 were included in the taxa for HW > other (33, 16, 0, 0), 10 for other > NW (0, 10, 0, 0), 34 for other > HW (11, 17, 5, 1), and 3 for NW > other (2, 0, 1, 0). The probabilities of MetaCyc classes between the HW > other and other > HW comparisons were significantly different according to Fisher's exact test (p < 0.01). In the HW > other comparison, aromatic compound degradation was the most abundant (the total number of correlations was 26) degradation/utilization/assimilation function. In the other > HW comparison, cofactor, carrier, and vitamin biosynthesis associated with biosynthesis of cytoplasmic membrane were the most abundant (the total number of correlations was 8) biosynthesis functions.

## Discussion

We examined the ruminal *microbial and chloroplast composition* of wild sika deer using next-generation sequencing and subsequent analysis.

### The dominant ruminal microbial taxa in Japanese sika deer

Our results indicated that the dominant microbes in Japanese sika deer are similar to those in other ruminants, especially wild ruminants. The dominant genera of Bac taxa (Fig. 4 and Supplementary Table S5) revealed by amplicon-seq in the present study were consistent with the ruminal microbiome reported previously in sika deer^16,17,19^ and related species (wild roe deer^13^, elk^14,18^, reindeer^20^, ruminants^6^). Of the previous reports, Henderson et al.^6^ reported the dominant core rumen bacteria of 32 species of ruminants and a total of 742 individual animals. The core bacteria were similar across all ruminants; the top mean relative abundances of bacterial groups in 60 deer were reported here to be almost 70% and were observed for *Prevotella*, unclassified Clostridiales, Bacteroidales, *Ruminococcaceae*, *Lachnospiraceae*, *Veillonellaceae*, and *Ruminococcus*.

In the analysis of the dominant Euk taxa (Fig. 5B and Supplementary Table S7), g_*Piromyces* and g_*Entodinium* were the dominant genera. This dominance has been reported in ruminants^6^, sika deer in Japan^10^ and China^17^, and red deer in New Zealand^32^. Henderson et al.^6^ reported that the abundances of the dominant core rumen protozoa in the deer were almost 40% for *Entodinium* and 10% for *Metadinium*, *Polyplastron*, and *Epidinium*. This percentage for *Entodinium* is similar to our result for NS and NW but not HW. Of the 60 deer samples, five samples of Japanese sika deer (*Cervus nippon yesoensis*) obtained postmortem in January of the winter season in Hokkaido, Japan, were included in the report of Henderson et al.^6^. Similar to our results for HW, the average number of protozoa sequence reads was less than 10, while that of bacteria was almost 10 thousand.

Thus, we considered the results of our study to be informative for understanding the diversity of ruminal microbes and chloroplasts associated with different food habits developed over a long history of adaptation to natural habitats in Japanese sika deer. In addition, they could be useful for understanding the rumen composition of livestock ruminants.

### Genera enriched in the rumen among the groups

The groups differed in ruminal microbe and plant composition, with an order from most to least diverse of NW, NS, and HW, as shown by alpha diversity metrics for Euk and Chl and beta diversity distance for Bac and Euk (Figs. 1 and 3, and Tables 1 and 2).

Of the Bac taxa, the LEfSe technique revealed that g_*Prevotella* 1 and *Succinivibrionaceae* were enriched in NS (Fig. 4A and Supplementary Table S7). *Prevotella ruminicola*, *P. albensis*, *P. brevis*, and *P. bryantii* have been reported to have expanded ruminal niches under starch-, pectin-, xylan-, and sugar-rich conditions^5^. It has been reported that *Prevotella* and *Succinivibrionaceae* are more abundant in animals fed diets containing concentrate than in those fed forage-rich diets^6^. These taxa may be major producers of propionate and the propionate precursor succinate and thus might be responsible for the greater levels of propionate formed under concentrate-rich diets^6^. It has been reported that the proportion of *Prevotella* and the ruminal concentration of ammonia decreased on day 28 after the start of cellulose-rich corn silage feeding in sika deer^16^. A positive association between *Prevotella* and the ruminal concentration of butyrate was also shown previously^16^. On the other hand, in the present study, cellulolytic bacteria such as *Lachnospiraceae* and *Ruminococcaceae*^33^ were enriched in HW (Fig. 7 and Supplementary Table S10). In particular, *R. flavefaciens* and *F. succinogenes*, enriched in HW, are well-studied cultivable bacteria^5,34,35^. Consistent with our results, it has been reported that the sika deer rumen flora contains a higher proportion of *R. flavefaciens* in winter than in summer at Shiretoko^11^. Under winter conditions in the same habitat, a shortage of food corresponds to a decrease in ruminal concentrations of total VFAs, propionate, butyrate, ammonia and mineral contents in sika deer^10,36^. Given the accordance between the results of previous reports and those of the present study, it is hypothesized that the amounts of nutrients in the rumen, such as cellulose, ammonia and total VFAs, are associated with the proportion of Bac taxa enriched in Nagano and Hokkaido, such as *Prevotella* and cellulolytic bacteria, respectively (Fig. 7 and Supplementary Table S10).

Among Euk taxa, the relative abundance of *Entodinium* in HW was markedly low in comparison to NW and NS (Fig. 8 and Supplementary Table S11). Our results are consistent with the fact that *Entodinium* density in winter decreases to 1/10 of that in summer at Shiretoko^10^. In winter, due to the limited availability of deciduous broadleaf tree leaves, sika deer eat foods such as ground plants, bark, twigs, and lichens, which are less abundant and have lower-quality crude protein than tree leaves^10^. The sika deer in HW therefore could be considered adapted to utilizing low-quality diets because rumen protozoa have been reported to make minor contributions to fiber digestion^10^.

The whole transcriptomes of *Entodinium caudatum* have been reported using RNA-Seq analysis^37^. *E. caudatum* is considered to prefer starch since it has more transcripts involved in the use of starch than in the use of cellulose and hemicellulose. In addition, *E. caudatum* ferments sugars to VFAs. In the present study, *C. glandium* was enriched in NW (Fig. 8 and Supplementary Table S11). In winter in Nagano, sika deer might prefer to eat acorns because weevil larvae often grow in acorns over the winter^38^. Acorns themselves are rich in starch and lipids^39^. The ruminal concentration of total VFAs was reported to be higher in summer and autumn than in winter^10^. Rumen protozoa affect the meat quality of ruminant mammals in terms of fatty acid composition. It has been reported that rumen protozoan membranes contain high concentrations of conjugated linoleic acids (CLAs) and vaccenic acid^40^, and the total ciliate number in the rumen has a positive correlation with cis-9, trans-11 CLAs and vaccenic acid depositions in lamb meat^41^. These findings suggested that rumen *Entodinium* could play an important role in storing nutrients in the muscle for severe winter seasons after deer consume fallen leaves and acorns in autumn.

Among the Euk taxa, the anaerobic fungi f_*Neocallimastigaceae* and g_*Piromyces,* well-established species with utility for lignocellulose bioprocessing^42^, were more enriched in HW than in NW and NS (Fig. 8 and Supplementary Table S11). It has been reported that the transcripts of biomass-degrading enzymes are repressively controlled by bacteria via glucose stimulation^42^. The comparison of transcriptomes among anaerobic and aerobic fungi and rumen and nonrumen bacteria showed that *P. rhizinflata* had almost twice as many putative pectinases as the other anaerobic lignocellulolytic fungi^43^. These putative pectate lyases were conserved among the anaerobic fungi, whereas these enzymes were much less commonly found in the other groups of organisms, particularly bacteria^43^. Under winter conditions at Hokkaido, nutritional shortages could be required for *Piromyces* to degrade pectin within the bark. In our results, putative glycolysis- and glycogenesis-estimated DE transcripts were observed in the rumen bacteria of HW (Table 3 and Fig. 10). It was considered that the number and function of rumen lignocellulolytic fungi might be regulated by cellulolytic bacteria to overcome severe winter conditions.

### Seasonal differences in the food habits of wild sika deer in Japan

The alpha and beta diversity metrics of Chl taxa were different among NW, NS, and HW, suggesting seasonal changes in the food habits of sika deer in Japan (Figs. 1, 2, 3 and Tables 1 and 2). The present study indicates that the NW diet is enriched in broadleaf trees (Fagales, *Morus*, and *Ilex*), which bear many acorns and winter buds (Fig. 6 and Supplementary Table S9), whereas the NS diet is enriched in spring- to summer-flowering deciduous broadleaf trees (*Populus*, *R. pseudoacacia,* and *A. quinate)*, evergreen trees (*C. camphora, Fokienia)* and forbs (*P. tenella*) (Fig. 6 and Supplementary Table S9). The food habits of sika deer living close to Nagano have been described^24,25^. In winter to spring in the wildlife reserve area of Ashio, Tochigi, the predominant rumen contents of sika deer were culm sheaths, dead leaves, barks and twigs, conifers, and graminoids (from most to least prevalent)^24^. In autumn to winter at Mount Akagi, Gunma, the diet was composed of graminoids, dead leaves of broadleaf trees, nuts, and berries^25^. These results indicated that the sika deer in Nagano consume mostly broadleaf tree leaves and nuts during winter to spring.

In the present study, the rumen composition of HW was enriched in graminoids (Poales) and forbs (*Panax)* (Fig. 6 and Supplementary Table S9). In winter, at Shiretoko, Hokkaido, bark and twigs, graminoids, and leaves were the main foods in the rumen contents of sika deer^10^. In February, at Ashoro and Onbetsu, Hokkaido, the rumen contents were composed of broadleaf tree leaves, bark and twigs, graminoids and forbs^23^. Among the Poales, Sasa bamboo (*Sasa nipponica*) has been reported to be a predominant food species, especially in winter, for wild sika deer in Japan^10,23–25^. *S. nipponica* has evergreen leaves, and its nutritional content is moderate compared with that of other plants^23^. The Chl taxa enriched in HW indicated that Poales taxa are an important source of nutrients for sika deer in Hokkaido during severe winter periods with heavy snow.

### The associations among nutritional sources, fermentation, and rumen microbial and chloroplast composition

Given the discussion above, it is hypothesized that the rumen bacteria (e.g., *Lachnospiraceae* and *Ruminococcaceae*) and anaerobic fungi (*P. finnis*) enriched in HW prefer to grow in cellulose-rich and starch- and lipid-poor conditions, while abundant starch and lipids are fermented by *Prevotella, Succinivibrionaceae* and ciliates (e.g., *Entodinium*) to generate large energy stores. It is suggested that the rumen bacteria and anaerobic fungi in HW extract glucose and xylose from the fermentation of cellulose, hemicellulose and lignin while saving and using small energy sources. It has been reported that the number of rumen bacteria increases and that of *Entodinium* decreases in winter based on morphological examinations of deer^10,44^. Significant numbers of ruminal bacteria can be consumed by protozoa, resulting in an inverse relationship between protozoal and bacterial densities^5^. Total RNA-seq analysis revealed many glycolysis- and glycogenesis-estimated DE transcripts in the rumen in the HW > other comparison (Table 3). Furthermore, the majority of the taxa with differential expression that were highly associated with the predicted DE transcripts were also enriched in HW (Fig. 10). The MetaCyc pathways associated with aromatic compound degradation were correlated with the bacterial taxa identified in the HW > other category (Fig. 11). Aromatic compounds are secondary metabolites of lignin, so this result suggested that sika deer consume low-nutrient foods in HW^45^. Our results suggested that bacterial activity was enhanced under rumen conditions in HW. The decrease in protozoa indicated that the interaction of rumen bacteria with anaerobic fungi compensates for the presence of protozoa.

### Limitations

We could not examine fermentation parameters in the rumen or the quality of meat. Nagano sika deer and Hokkaido sika deer are different subspecies (*C. n. centralis* and *yessoensis*, respectively), and an effect of host genetic background on the rumen microbiome and metabolites in sika deer and elk hybrids has been reported^15^. Further research is warranted to examine the roles that differences in the season and location play in structuring the rumen microbiome in the same genetic background, determining rumen physiological status and driving the quality of game meat from sika deer in Japan.

To reduce contamination, we employed RNAlater to stabilize the samples and to prevent bacterial multiplication^46^. However, environmental contamination could potentially have been present due to the lack of a negative control in our study. Although a negative control would not completely ensure the absence of possible environmental contamination in the samples^47^, bioinformatic tools such as DECONTAM would be helpful for identifying and removing contaminant-related DNA sequences in microbiome data via statistical classification procedures^47^. Additional studies using negative controls and DECONTAM could improve the accuracy of the profiling of taxa with low abundance.

### Correlation between total RNA-seq and amplicon-seq data

Strong correlations between the annotated DE transcripts and bacterial taxa with differential expression were observed in the present study (Fig. 10, and Supplementary Table S14). The correlated taxa with differential expression were consistent with the genera detected as enriched by the LEfSe technique, such as f*_*Lachnospiraceae (*E. ruminantium*)*,* g_*Lachnoclostridium* 10, g_*Lachnospiraceae* XPB1014 group, g_*Fibrobacter*, and g_*Ruminococcus* 1 in HW and g_*Prevotella 1* in NS (Supplementary Tables S7 and S14). The correlated flagellin genes could indicate that rumen bacteria have motility and can adhere to plant tissues (Fig. 10)^48^. The correlation with the EMP pathway, LDH, PTS, and sucrose catabolism could indicate that rumen bacteria utilize glucose, sucrose, and pyruvic acid for glycolysis, resulting in the production of VFAs (Fig. 10 and Supplementary Table S14)^49,50^. Biological information converted from annotated transcripts has been used to study the seasonal prevalence of functional marine microorganisms^27^. The large set of gene expression data from the total RNA-seq analysis could indicate the accuracy of genomic data from amplicon-seq from different perspectives. One of the criticisms of total RNA-seq is that it is limited in the biological information provided by annotated transcripts. In this study, only 119 of the 397 DE transcripts provided information by a homology search using BLAST + against the Swiss-Prot database. This limitation is decreasing over time as the amount of biological information in the database continues to grow.

In this study, MetaCyc pathway abundances revealed by PICRUSt2 analysis were used to analyze correlations with amplicon-seq data^51^. PICRUSt2 predicts the functional potential of the bacterial community using 16S rRNA marker gene data. One of the limitations of this amplicon-based approach is that PICTRUSt2 cannot provide sufficient resolution to distinguish strain-specific functionality^51^. In this study, the biological function associated with amplicon-seq results differed between the total RNA-seq and PICRUSt2 approaches. Total RNA-seq may be superior to amplicon-seq when information is required from a live subject, such as the detection of the EMP glycolysis pathway and locomotion for rumen bacteria. Moreover, it has been reported that both approaches show varying results^30,52^, so a combination of different approaches is desirable to permit an evaluation of the accuracy of rRNA data.

## Conclusion

In the present study, using 16S and 18S rRNA amplicon-seq and total RNA-seq analysis, we revealed differences in population-level microbiome and transcriptome diversity between seasons (spring/winter) and locations (Nagano/Hokkaido) in wild sika deer in Japan. Diversity was reflected by alpha diversity metrics for Euk and beta diversity metrics for Bac and Euk. The LEfSe results showed the enrichment of specific Bac and Euk taxa, and a difference in food habits was revealed by the enriched Chl taxa. Previous reports and the results of the present study provide insight into the relationship between the ruminal community (bacteria, fungi, and protozoa) and food habits (limited vs. abundant feeding conditions). The strong correlation between the results of amplicon-seq and total RNA-seq analysis showed the accuracy of the present data and their ability to provide information about the live cell function (locomotion ability and glycolysis) of ruminal bacteria. These data are useful not only for understanding the nutritional status of wild sika deer in Japan but also for understanding microbial community dynamics during the fermentation process in ruminants.

## Materials and methods

### Animals and sampling (Supplementary Tables S1 and S2)

Twenty-nine (16 male and 13 female) rumen samples were obtained from adult sika deer, 24 living on Mount Asama (*Cervus nippon centralis*), Nagano, and 5 living in Shiretoko, Hokkaido (*C. n. yessoensis*). Twelve samples from Nagano were collected in the winter season between December 2019 and January 2020 (referred to as NW), and the other 12 samples were collected in the spring season during April 2020 (referred to as NS). All 5 Hokkaido samples were collected in the winter season during February 2019 (referred to as HW). The mean temperature (maximum/minimum) recorded by the Japan Meteorological Agency was 7.0/− 4.0 °C in December–January in Nagano and 16.0/− 2.0 °C in April in Nagano and was − 2.2/− 9.6 °C in February–March in Hokkaido. The monthly snow depths were 16 cm in April 2019, 27 cm in December 2019, and 26 cm in January 2020 in Karuizawa, Nagano (36′ N, 138′ E), and 123 cm in February 2019 in Utoro, Hokkaido (44′ N, 145′ E), which were close to the sampling area. In Hokkaido, hunted sika deer were immediately dressed, and their rumen samples were collected in the field. In Nagano, the culled deer were moved to a slaughter facility operated by the Komoro city government. We used RNAlater (Thermo Fisher Scientific Japan, Tokyo, Japan) as a stabilizer to examine both DNA and RNA^46^. One milliliter of rumen juice was carefully placed into a sterile 15 ml conical tube filled with 9 ml of RNAlater solution by a disposable, individually packaged, clean plastic spoon. Then, the tubes were sent to the laboratory at Azabu University and stored at 4 °C within 3 days. The tubes were kept in a − 80 °C freezer until DNA and RNA preparation.

### Animal ethics

The collection of rumen samples from sika deer was approved by the ethics committee of animal experiments at Azabu University (approval 150917-1) and conducted in accordance with the ethical standards of The Mammal Society of Japan (http://www.mammalogy.jp/en/guideline.pdf) in the appropriate way as reported^2^. Permissions to conduct deer culling in this study were obtained from Japanese government according to the “Wildlife Protection and Proper Hunting Act” of the Ministry of the Environment. This study was performed accordance with ARRIVE guidelines. Permission numbers and the permitters are described in Supplementary Table S1 and S2.

### DNA and RNA preparation

One hundred microliters of the RNAlater-fixed rumen content suspension was collected by a pipet tip. The suspension was centrifuged (21,130×*g*, 1 min), and then the supernatant was discarded. The remaining pellet was used for DNA and RNA extraction with a DNeasy PowerSoil Kit (QIAGEN, Venlo, Netherlands) and RNeasy PowerBiofilm Kit (QIAGEN), respectively. The extracted DNA and RNA were eluted and dissolved in 100 µl of water. DNA and RNA concentrations were measured by a Qubit DNA and RNA HS Kit (Thermo Fisher, Waltham, MA, USA).

### Construction and quantification of DNA and RNA libraries

The prepared DNA was used for the construction of 16S and 18S rRNA gene sequence libraries using the “16S Metagenomic Sequencing Library Preparation” protocol (Illumina, San Diego, CA, USA) with some modifications. Briefly, “KOD plus” (TOYOBO, Osaka, Japan) PCR enzyme was used with standard cycle conditions (98 °C for 2 min; 30 cycles of 98 °C for 15 s, 55 °C for 45 s and 68 °C for 1 min; and then 68 °C for 6 min) and a standard reaction mixture (1.5 mM MgSO_4_, 0.2 mM dNTP, 1 unit/50 µl KOD plus, and 0.2 pmol/µl primers) for the first amplification. The first forward PCR primer targeting 16S rRNA (V3-V4 region) was 341F (5′-TCG TCG GCA GCG TCA GAT GTG TAT AAG AGA CAG CCT ACG GGN GGC WGC AG-3′), and the reverse primer was 805R (5′-GTC TCG TGG GCT CGG AGA TGT GTAT AAG AGA CAG GAC TAC HVG GGT ATC TAA TCC-3′)^53^. The first forward PCR primer targeting 18S rRNA (V4 region) was modified TAReuk454FWD1 (5′-TCG TCG GCA GCG TCA GAT GTG TAT AAG AGA CAG CCA GCA SCY GCG GTA ATT CC-3′), and the reverse primer was TAReukREV3 (5′-GTC TCG TGG GCT CGG AGA TGT GTA TAA GAG ACA GAC TTT CGT TCT TGA TYR A-3′)^54^. The first PCR products were purified using Agencourt AMPure XP (Beckman Coulter, Brea, CA, USA) and eluted with 50 µl of water. A second PCR was performed using the primers of a Nextera XT index kit (Illumina). The cycle conditions were 98 °C for 2 min; 12 cycles at 98 °C for 15 s, 55 °C for 15 s, and 68 °C for 1 min; and then 68 °C for 6 min. The concentration of PCR products was measured by a Qubit DNA HS Kit (Thermo Fisher) and equalized by water.

A total RNA-seq library was constructed using a SMARTer stranded RNA-seq kit (Clontech, San Francisco, CA, USA). PCR amplification was repeated for 12 cycles according to the manufacturer’s instructions. PCR products were eluted with 15 µl of water, measured with a Qubit DNA HS Kit and equalized with water.

Quantification of both pooled sequencing libraries was performed by qPCR using the Library Quantification Kit for Illumina sequencing platform (KAPA Biosystems, Boston, USA). The concentration was adjusted to 2.0 nM. Paired-end reads 250 base pairs (bp) in length with 5% PhiX spike were loaded for Illumina MiSeq sequencing.

### Sequence analysis

The obtained 16S and 18S rRNA gene sequence reads were analyzed using the Quantitative Insights Into Microbial Ecology (QIIME2) pipeline^55^ with DADA2^56^ for quality control. The generated OTUs were assigned using a naïve Bayes classifier for annotation^57^. Each target region was specified by primer sequences to strains in the naïve classifier with silva132_99.fna of the Silva database, release 132^58^. Annotation was performed on taxonomy_7_levels.txt in the same database. When the identified plant species were not distributed in Japan, genus-level taxa were reported in the manuscript (raw data are shown in Supplementary Table S9).

Total RNA-seq analysis was performed according to the previously reported ARI-seq analysis pipeline^59^. In detail, the obtained total RNA-seq reads were trimmed by Trimmomatic-0.39^60^ with the option “ILLUMINACLIP: TruSeq_LT_HT.fa:5:30:7 MINLEN:100 HEADCROP:6 LEADING:20 TRAILING:20”. PhiX sequences were removed by the USEARCH 11.0.667-filter_phix option^61^. Low-complexity filtering was performed with the USEARCH 11.0.667 -filter_lowc option. The cleaned reads were assembled by Trinity v2.11.0 with a minimum_contig_length of 500^62^. The reads were sorted into rRNA and non-rRNA reads using SortMeRNA^63^ with the reference sequences of silva-arc-16s-id95.fasta, silva-bac-16s-id90.fasta, and silva-euk-18s-id95.fasta. The sorted rRNA reads were mapped against Trinity output (Trinity.fasta) by Bowtie2 v.2.3.5.1-linux-x86_64^64^ with options-U and -local. The resulting SAM files were transformed into bam files using SAMtools and then sorted. The sorted BAM files were used to calculate counts by eXpress v.1.5.1-linux_x86_64^65^. The count data were truncated with a custom script to remove reads with fewer than 10 observations. The expressed genes were annotated for the expected ORF sequence by TransDecoder software. The resulting expected ORF sequences were annotated by BLAST + against the Swiss-Prot database.

### Statistical analysis

In amplicon-seq analysis, based on the taxonomy generated for microbes, we excluded reads originating from archaea, chloroplasts, and host mitochondria. The reads from the chloroplasts were used for ruminal plant taxa. We removed singleton features and elements with fewer than 10 reads. The filtered feature tables and computed relative abundance per taxonomic level values were used for diversity analyses. The alpha and beta diversity metrics and their group significance were calculated using the QIIME 2 pipeline^55^. Rarefaction-curve analyses of the obtained data were performed to estimate the completeness of taxonomic community sampling. Subsequently, alpha diversity metrics (Shannon’s diversity index, observed OTUs, Faith’s phylogenetic diversity, and Pielou’s evenness) were examined to detect differences in the number of species and species richness per sample, and statistical significance among the groups was computed using the Kruskal–Wallis (KW) test. The effects of the other variables (sex and age) among groups were examined using generalized linear models with R functions^66^. Beta diversity metrics (Jaccard, Bray–Curtis, unweighted UniFrac, and weighted UniFrac distances) were visualized using principal coordinate analysis (PCoA) with Emperor, and significant differences among and within the groups were detected using ANOSIM. The Benjamini–Hochberg false discovery rate (FDR) correction was then used to identify each metric differently represented between groups. p- and q-values < 0.05 were considered statistically significant. The LEfSe approach^67^ was implemented to identify the microbial taxa that were significantly associated with the groups. The LEfSe algorithm consisted of a KW rank sum test for detecting differences between classes and LDA for detecting differences in the relevant features. The parameters were set at p = 0.05 and LDA score = 3.0 for the computation.

All components of the total RNA-seq analysis were performed using R functions^66^. Tag Count Comparison (TCC) baySeq^68^ was used for normalization and differential expression analysis of the multigroup RNA-seq count data. The TCC package was generated from original TbT methods (TMM-baySeq-TMM pipeline), consisting of a combination of the trimmed mean of M values (TMM) normalization^69^ in edgeR^70^ and DE gene detection in baySeq^71^. In this strategy, normalization of count data and DEG estimation are iterated to avoid false positives; we repeated this cycle three times in this study^72^. Transcripts were considered DE between groups at a FDR (q-value) lower than 0.05.

PICRUSt2 analysis was performed with dada2 output during QIIME2 analysis^51^. Representative sequences and feature tables were transformed by QIIME2 and then used for PICRUSt2 analysis. The default full pipeline command “picrust2-pipeline.py” was used with default parameters for this analysis. A sequence table normalized by predicted 16S copy number abundances was used as a predicted MetaCyc pathway abundance table for further correlation analysis.

The Pearson correlation coefficients between amplicon-seq data, transcriptome data and MetaCyc pathway data were calculated using the “cor” function of R^66^. The obtained matrix was used to draw a heatmap with the “gplots” package of R. In this heatmap, we visualized correlations with a coefficient between 0.7 and 1.0.

## Supplementary Information

Supplementary Tables.

Supplementary Figures.

## Data Availability

The datasets generated and/or analyzed during the current study are available in the DDBJ Sequence Read Archive repository, accession number DRA011262 for 16S the rRNA gene sequence library, DRA011263 for the 18S rRNA gene sequence library, and DRA011264 for the total RNA-seq library.
