# Author Correction: Seasonal and geographical differences in the ruminal microbial and chloroplast composition of sika deer (*Cervus nippon*) in Japan

**DOI:** 10.1038/s41598-022-12520-x

**Published:** 2022-05-17

**Authors:** Shinpei Kawarai, Kensuke Taira, Ayako Shimono, Tsuyoshi Takeshita, Shiro Takeda, Wataru Mizunoya, Yumiko Yamazaki, Shigeharu Moriya, Masato Minami

**Affiliations:** 1 Laboratory of Small Animal Clinics, Veterinary Teaching Hospital, Azabu University, 1‑17‑71 Fuchinobe, Chuo‑ku, Sagamihara, Kanagawa 252‑5201 Japan; 2 Laboratory of Parasitology, School of Veterinary Medicine, Azabu University, 1‑17‑71 Fuchinobe, Chuo‑ku, Sagamihara, Kanagawa 252‑5201 Japan; 3 Plant Ecology Laboratory, Department of Biology, Faculty of Science, Toho University, 2‑2‑1 Miyama, Funabashi‑shi, Chiba 274‑8510 Japan; 4 Agriculture and Forestry Division, Komoro City Office, 3‑3‑3, Aioicho, Komoro, Nagano 384‑8501 Japan; 5 Laboratory of Food Science, Department of Animal Science and Biotechnology, School of Veterinary Medicine, Azabu University, 1‑17‑71 Fuchinobe, Chuo‑ku, Sagamihara, Kanagawa 252‑5201 Japan; 6 RIKEN BDR, Kobe, Hyogo 650‑0047 Japan; 7 RIKEN CSRS, Yokohama, Kanagawa 230‑0045 Japan; 8 Laboratory of Wildlife Ecology and Conservation, Department of Animal Science and Biotechnology, School of Veterinary Medicine, Azabu University, 1‑17‑71 Fuchinobe, Chuo‑ku, Sagamihara, Kanagawa 252‑5201 Japan; 9 Center for Human and Animal Symbiosis Science, Azabu University, 1‑17‑71 Fuchinobe, Chuo‑ku, Sagamihara, Kanagawa 252‑5201 Japan

Correction to: *Scientific Reports* 10.1038/s41598-022-09855-w, published online 15 April 2022

The Supplementary Information section published with this Article contained an error where the Supplementary Figures were omitted. In addition, in the Supplementary Tables the cells N8 in Table S1 and E4 in Table S14 were incorrectly highlighted.

These errors have now been corrected in the Supplementary Information section that accompanies the original Article.

