# Supplementary material for: Seasonal and geographical differences in the ruminal microbial and chloroplast composition of sika deer (Cervus nippon) in Japan
Source: Sci Rep. 2022 Apr 15;12:6356. doi: 10.1038/s41598-022-09855-w (PMC9012793; doi:10.1038/s41598-022-09855-w)
Supplement: Supplementary file 2 — Supplementary Figures. [file 41598_2022_9855_MOESM2_ESM.pptx]

## Slide 1
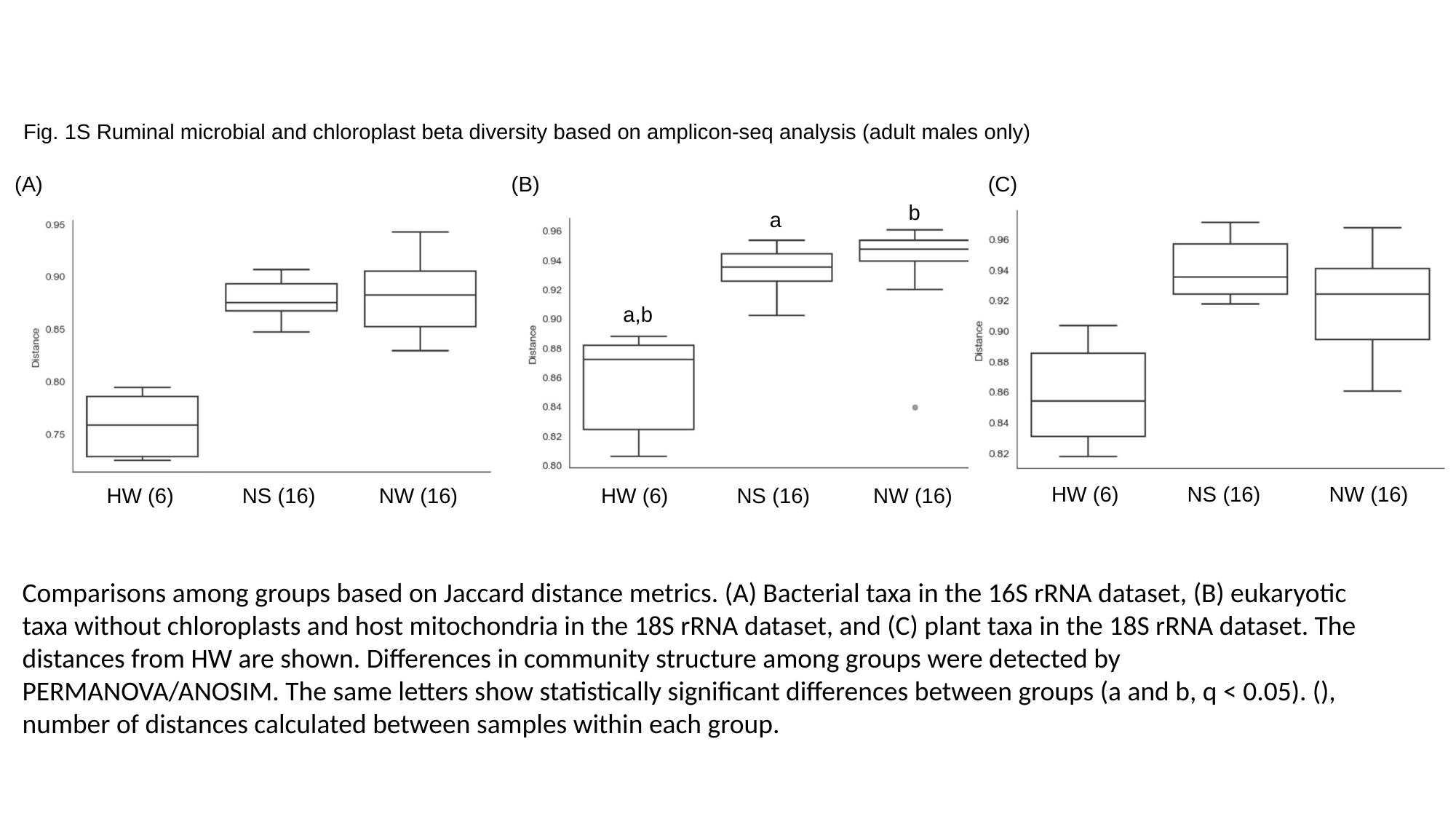

Fig. 1S Ruminal microbial and chloroplast beta diversity based on amplicon-seq analysis (adult males only)
(A)
(B)
(C)
b
a
a,b
HW (6)
NS (16)
NW (16)
HW (6)
NS (16)
NW (16)
HW (6)
NS (16)
NW (16)
Comparisons among groups based on Jaccard distance metrics. (A) Bacterial taxa in the 16S rRNA dataset, (B) eukaryotic taxa without chloroplasts and host mitochondria in the 18S rRNA dataset, and (C) plant taxa in the 18S rRNA dataset. The distances from HW are shown. Differences in community structure among groups were detected by PERMANOVA/ANOSIM. The same letters show statistically significant differences between groups (a and b, q < 0.05). (), number of distances calculated between samples within each group.

## Slide 2
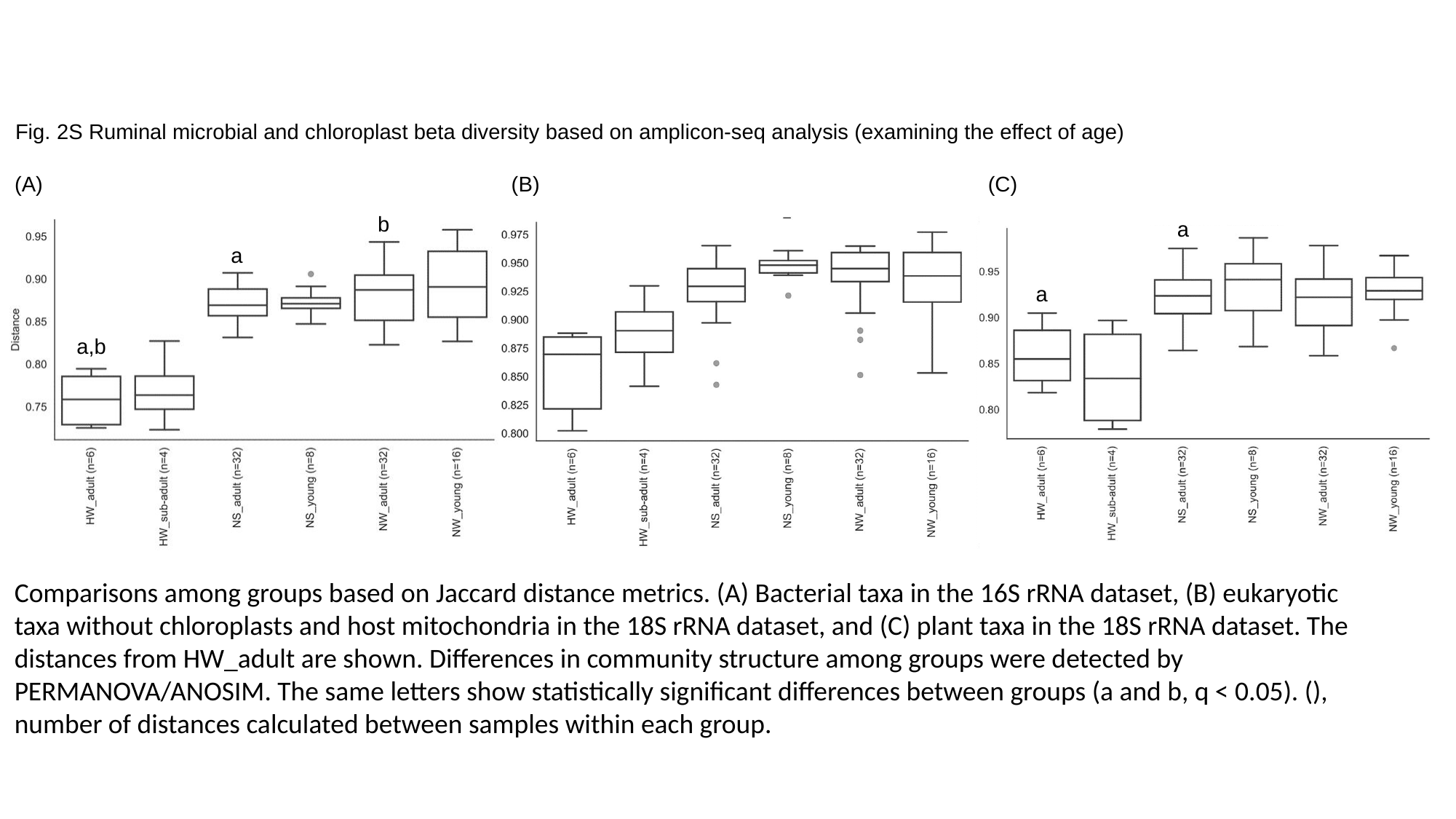

Fig. 2S Ruminal microbial and chloroplast beta diversity based on amplicon-seq analysis (examining the effect of age)
(A)
(B)
(C)
b
a
a
a
a,b
Comparisons among groups based on Jaccard distance metrics. (A) Bacterial taxa in the 16S rRNA dataset, (B) eukaryotic taxa without chloroplasts and host mitochondria in the 18S rRNA dataset, and (C) plant taxa in the 18S rRNA dataset. The distances from HW_adult are shown. Differences in community structure among groups were detected by PERMANOVA/ANOSIM. The same letters show statistically significant differences between groups (a and b, q < 0.05). (), number of distances calculated between samples within each group.

## Slide 3
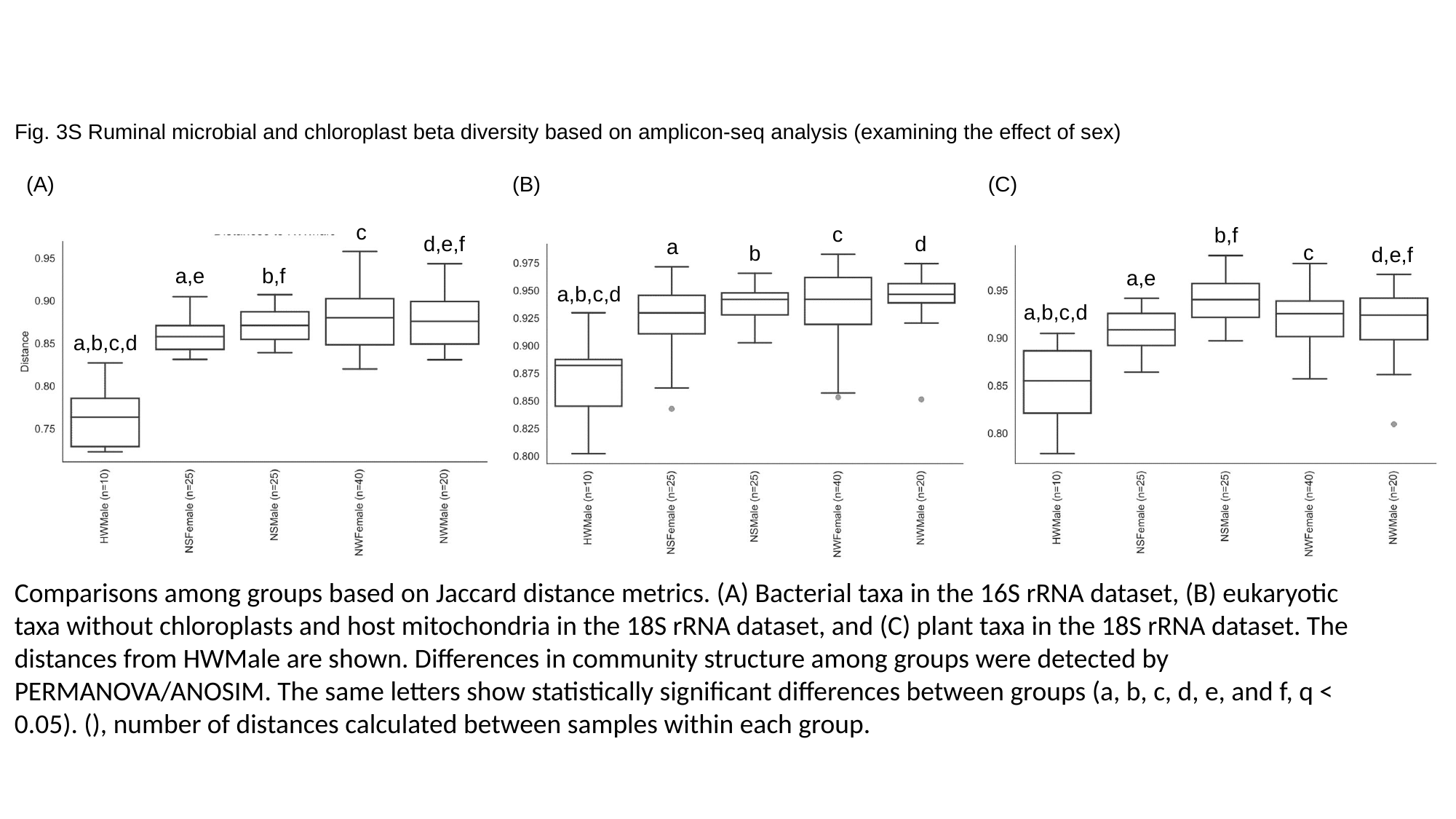

Fig. 3S Ruminal microbial and chloroplast beta diversity based on amplicon-seq analysis (examining the effect of sex)
(A)
(B)
(C)
c
c
b,f
d,e,f
d
a
c
b
d,e,f
b,f
a,e
a,e
a,b,c,d
a,b,c,d
a,b,c,d
Comparisons among groups based on Jaccard distance metrics. (A) Bacterial taxa in the 16S rRNA dataset, (B) eukaryotic taxa without chloroplasts and host mitochondria in the 18S rRNA dataset, and (C) plant taxa in the 18S rRNA dataset. The distances from HWMale are shown. Differences in community structure among groups were detected by PERMANOVA/ANOSIM. The same letters show statistically significant differences between groups (a, b, c, d, e, and f, q < 0.05). (), number of distances calculated between samples within each group.
